# Expanding the frontiers of electrocatalysis: advanced theoretical methods for water splitting

**DOI:** 10.1186/s40580-024-00467-w

**Published:** 2025-01-24

**Authors:** Seong Chan Cho, Jun Ho Seok, Hung Ngo Manh, Jae Hun Seol, Chi Ho Lee, Sang Uck Lee

**Affiliations:** 1 School of Chemical Engineering, Sungkyunkwan University, Suwon, 16419 Republic of Korea; 2 Artie McFerrin, Department of Chemical Engineering and Texas A&M Energy Institute, Texas A&M University, College Station, TX 77843 USA

**Keywords:** Water splitting reaction, Electrocatalyst, Hydrogen evolution reaction (HER), Oxygen evolution reaction (OER), Density functional theory (DFT), Machine learning (ML)

## Abstract

Electrochemical water splitting, which encompasses the hydrogen evolution reaction (HER) and the oxygen evolution reaction (OER), offers a promising route for sustainable hydrogen production. The development of efficient and cost-effective electrocatalysts is crucial for advancing this technology, especially given the reliance on expensive transition metals, such as Pt and Ir, in traditional catalysts. This review highlights recent advances in the design and optimization of electrocatalysts, focusing on density functional theory (DFT) as a key tool for understanding and improving catalytic performance in the HER and OER. We begin by exploring DFT-based approaches for evaluating catalytic activity under both acidic and alkaline conditions. The review then shifts to a material-oriented perspective, showcasing key catalyst materials and the theoretical strategies employed to enhance their performance. In addition, we discuss scaling relationships that exist between binding energies and electronic structures through the use of charge-density analysis and *d*-band theory. Advanced concepts, such as the effects of adsorbate coverage, solvation, and applied potential on catalytic behavior, are also discussed. We finally focus on integrating machine learning (ML) with DFT to enable high-throughput screening and accelerate the discovery of novel water-splitting catalysts. This comprehensive review underscores the pivotal role that DFT plays in advancing electrocatalyst design and highlights its potential for shaping the future of sustainable hydrogen production.

**Graphical Abstract:**

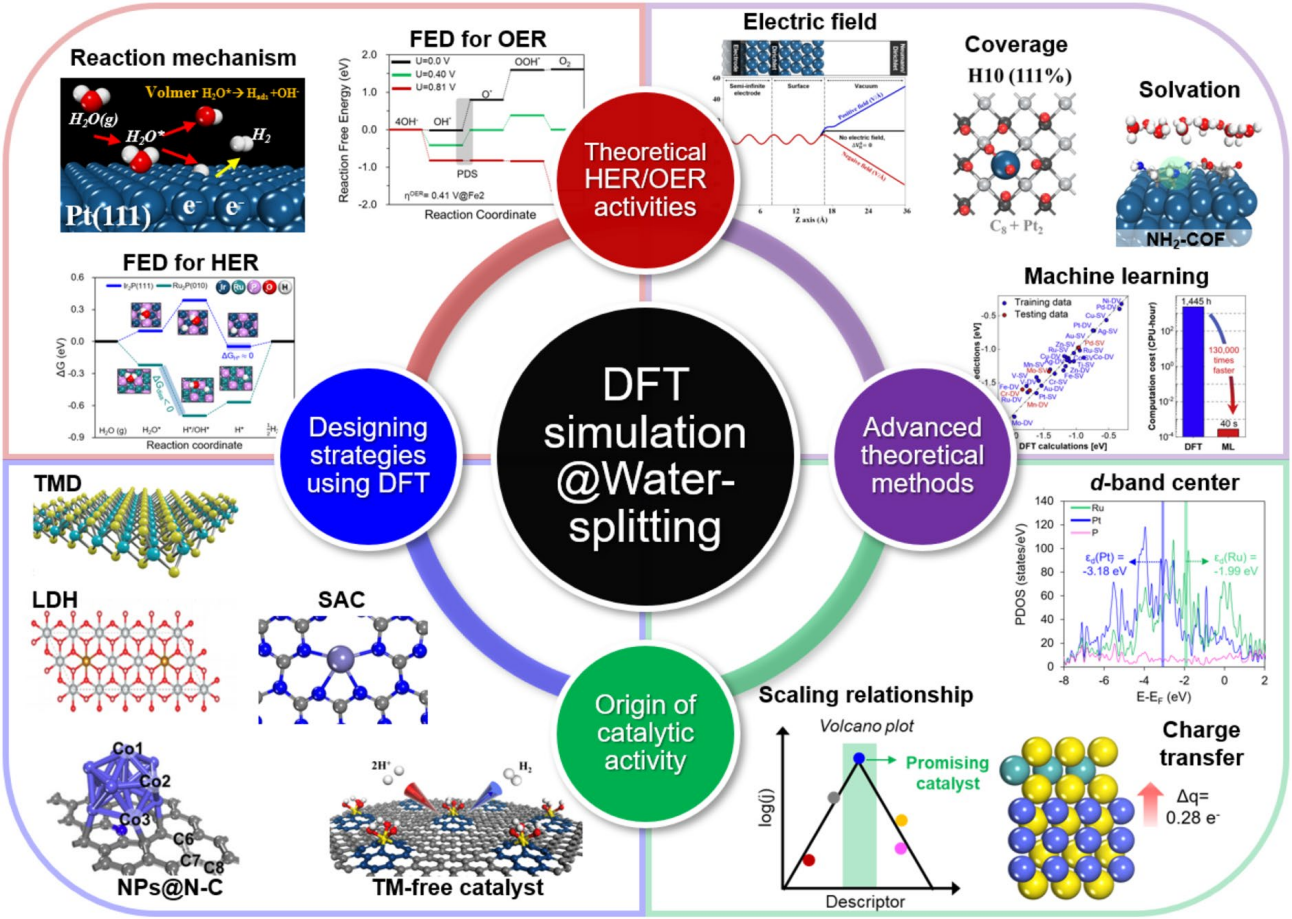

## Introduction

**Fig. 1:**
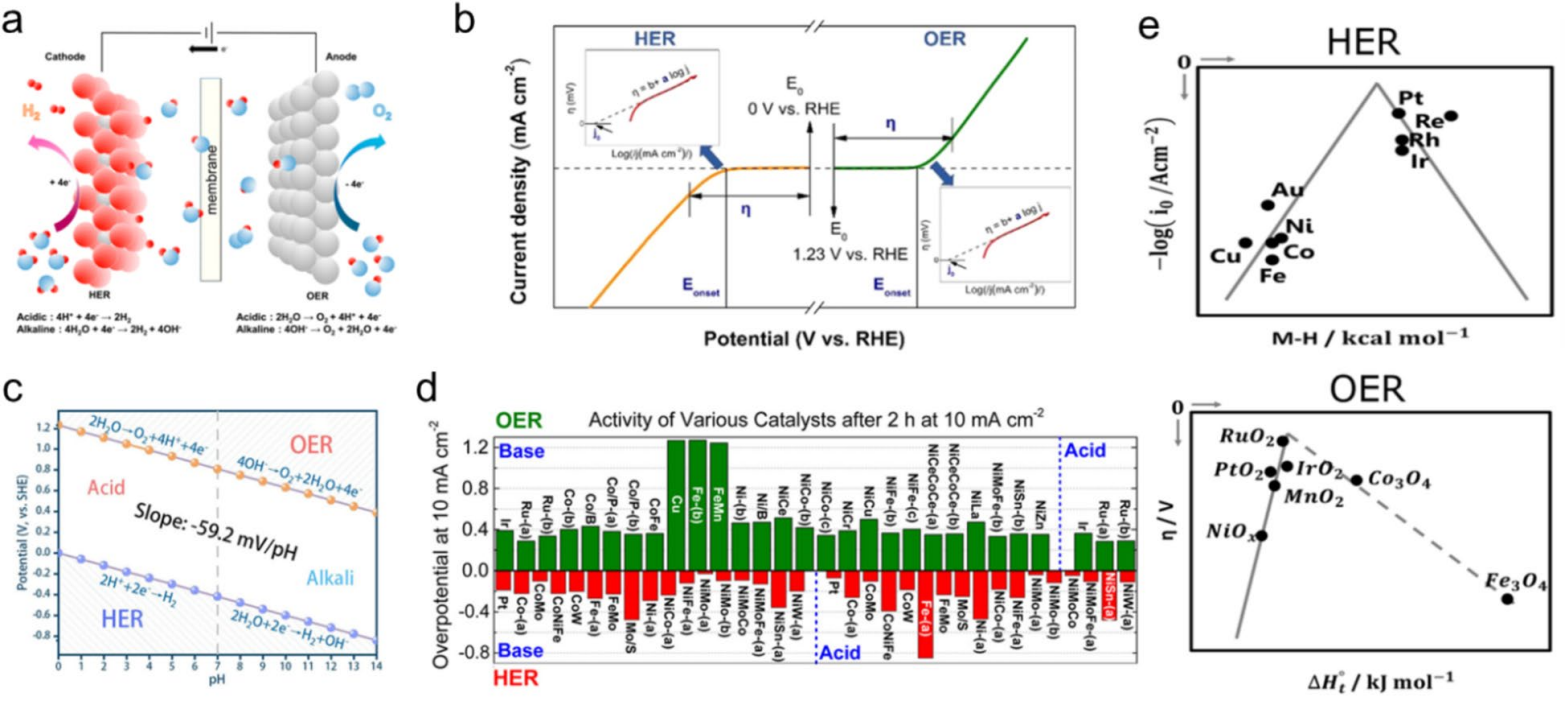
**a** Schematic depicting electrochemical water splitting on electrodes. **b** HER/OER current densities as functions of electrode potential, with corresponding HER (left) and OER (right) polarization curves. **c** Pourbaix diagram and HER/OER reaction processes showing potential and pH dependences. **d** Experimentally observed overpotentials required to achieve a current density of 10 mAcm^−2^ by various HER or OER electrocatalysts in basic and acidic solutions, respectively. **e** Volcano plots of exchange current density plotted against metal-H binding energy for the HER, and OER overpotential versus enthalpy of the oxide transition. (Figure panels are reproduced with permission [3, 4, 6–8])

The development of eco-friendly and sustainable energy sources is paramount for addressing global environmental challenges. Among various alternatives, the use of hydrogen gas (H_2_) as a source of energy is noteworthy owing to its substantial long-term energy-storage potential and its high energy density compared to other energy resources. Recent advances in clean hydrogen production have aimed to reduce CO_2_ emissions from traditional fossil-fuel-based steam-reforming processes [1, 2]. Electrochemical water electrolysis, which is a primary method for producing clean hydrogen, involves the hydrogen evolution reaction (HER) at the cathode and the oxygen evolution reaction (OER) at the anode, with both occurring at an equilibrium potential of 1.23 V (Fig. 1a, b) [3–5]. However, overcoming the energy barriers associated with these reactions requires substantial overpotentials that necessitate the development of highly active and stable electrochemical catalysts. The potential difference of 1.23 V between the HER and OER is independent of pH; hence, water-splitting catalyst research is feasible in both acidic and alkaline environments (Fig. 1c, d) [6, 7].

Precious transition-metal (TM) systems, such as Pt for the HER and RuO_2_ (or IrO_2_) for the OER, are known for their exceptional catalytic activities that are ascribable to their optimal adsorbate-binding affinities that follow the Sabatier principle, namely that binding affinity must be optimally balanced to ensure efficient catalysis—neither too strong nor too weak—thereby enabling effective reactant adsorption and product desorption. This optimal binding affinity is often depicted in volcano plots that show relationships between catalytic activity and binding strength (Fig. 1e) [8–10]. Although precious TMs exhibit impressive catalytic properties, their high costs pose significant challenges for large-scale hydrogen production. Consequently, researchers are increasingly directing their attentions to identifying cost-effective, Earth-abundant alternatives that can match the performance of traditional catalysts.

To address the abovementioned challenges, density functional theory (DFT) simulations have become indispensable for rationally designing catalysts by offering atomic-level insight into the reaction mechanisms and electronic structures responsible for catalytic activity. Because DFT enables new materials to be predicted and optimized under various reaction conditions, this approach not only accelerates the discovery of Earth-abundant catalysts but also provides valuable guidance for improving HER and OER activities in the absence of precious TMs. Hence, DFT is expected to play a crucial role in improving the performance of sustainable catalysts for large-scale hydrogen production.

Building on this principle, this review provides a comprehensive overview of the use of DFT in identifying and optimizing promising electrocatalysts. We explore structure–property relationships, particularly in nanostructured environments, and highlight geometric and electronic factors that influence catalytic performance. Additionally, this review discusses how DFT-driven approaches can be extended using advanced techniques, such as incorporating solvation effects, applied potentials, and machine learning (ML), as examples, to further accelerate the discovery and development of efficient electrocatalysts.

## DFT approaches in electrochemical HER and OER studies

### Roles played by DFT simulations in water-splitting catalysis

**Fig. 2:**
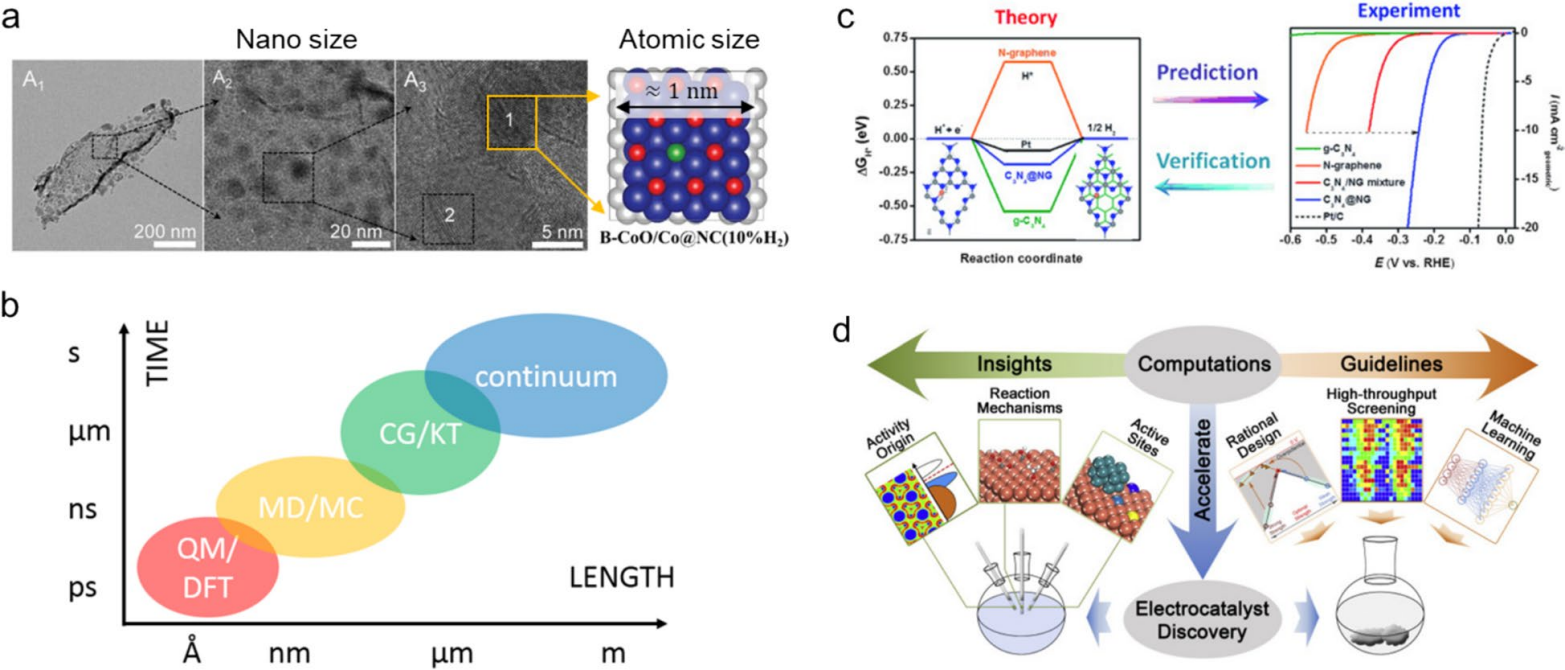
**a** Schematic depicting the size-dependent structural perspective associated with TEM and a theoretical catalyst model for electrochemical water splitting. **b** Length as a function of time scale for various modeling and simulation approaches. *QM* quantum mechanics, *DFT* density functional theory, *MD* molecular dynamics, *MC* Monte Carlo, *CG* coarse grain MD, *KT* kinetic theories. **c** Relationship between theory and experiment for water splitting based on simulated free-energy profiles and LSV curves acquired for the HER. **d** Schematic depicting the role played by computational approaches in accelerating electrocatalyst discovery by providing insight and guidelines through the use of DFT simulations. (Figure panels are reproduced with permission [11, 14, 19, 20])

A profound understanding of structure–property relationships is indispensable when optimizing electrocatalyst designs for the HER and OER, which hinges on chemical interactions between adsorbates and the catalyst surface, which are the primary determinants of catalytic behavior. Therefore, probing surface structures at the nanoscale level is crucial. Such investigations provide atomic-level insight that is paramount for uncovering the origins of catalyst performance, thereby enabling the underlying catalytic mechanisms to be elucidated and energy barriers and catalyst activities and selectivities within their operational environments to be assessed (Fig. 2a) [11–13].

Theoretical simulations are essential given the inherent limitations of experimental methods for providing comprehensive structural information. These simulations employ a diverse array of techniques tailored to meet specific research requirements. Techniques involving quantum mechanics (QM), density functional theory (DFT), molecular dynamics (MD), Monte Carlo (MC) simulations, kinetic theory (KT), and continuum models are selected based on the relevant length and time scales required to effectively model the phenomena of interest (Fig. 2b) [14–16]. Among these, DFT is particularly noteworthy because it is capable of calculating system energies using electron-density approximations, and strikes a balance between accuracy and computational feasibility that is particularly suitable for modeling phenomena at the nanometer and ångström scales [17, 18].

DFT not only facilitates predicting the catalytic activities of yet-to-be synthesized catalyst candidates, but also enables the origins of observed catalyst performance to be elucidated. Such predictive capabilities are critical for bridging the gap between experimental results and theoretical insight, thereby fostering the development of superior catalysts (Fig. 2c) [19]. Novel catalyst design processes are accelerated by leveraging DFT simulations, which positively impacts both time and cost efficiencies (Fig. 2d) [20]. Furthermore, this approach is continuously being refined through the integration of more complex system models and the incorporation of machine learning techniques into DFT frameworks, which enhances the predictive accuracies and efficiencies of the simulations [21–23]. When dynamic phenomena, such as solvent interactions or time-dependent catalytic behaviors, become significant, molecular dynamics (MD) can capture atomic motions over time, though it often lacks the precision needed for electronic-level interactions. Ab initio molecular dynamics (AIMD), which integrates DFT principles, offers a more accurate but computationally intensive approach to studying such dynamic processes. While these multiscale techniques provide critical insights, DFT remains the foundation of theoretical electrocatalysis research, offering an optimal balance between computational efficiency and predictive accuracy across various catalytic systems.

### Evaluating HER activities under acidic and alkaline conditions

**Fig. 3:**
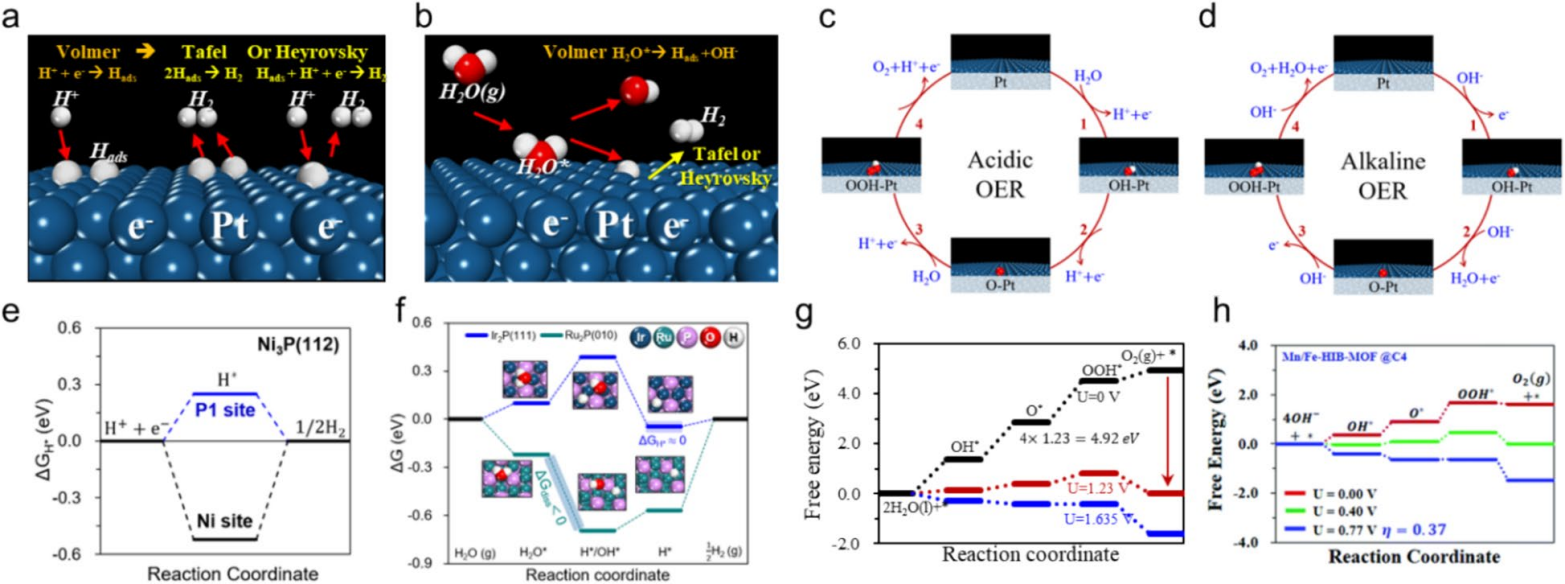
Electrochemical reaction mechanism for the HER under **a** acidic and **b** alkaline conditions, and for the OER under **c** acidic and **d** alkaline conditions. For HER, acidic conditions involve direct proton reduction, whereas alkaline conditions require H_2_O dissociation, introducing an additional energy barrier in the Volmer step. Calculated FEDs for the HER under **e** acidic and **f** alkaline conditions, and the OER under **g** acidic and **h** alkaline conditions on electrocatalysts. (Figure panels are reproduced with permission [33–37])

Evaluating HER activities in acidic or alkaline environments provides key insight into electrocatalytic water splitting, which fundamentally occurs through specific electrochemical processes on the catalyst surface. Initially, hydrogen adsorption at the active site, referred to as the Volmer step, sets the stage; here * and H* represent the active site and adsorbed hydrogen atom, respectively. This first step is followed by either the Heyrovsky or Tafel step, which facilitates the evolution of H_2_ gas, as depicted in Fig. 3a [24, 25]. These steps can be written as:

1 $$Volmer\,step:H^{ + } + e^{ - } + * \to H^{*}$$

2 $$Heyrovsky\,step:H^{ + } + e^{ - }+ H^{*} \to H_{2} + *$$

3 $$Tafel\,step:H^{*} + H^{*} \to H_{2} + *$$

While the Volmer, Heyrovsky, and Tafel steps describe HER mechanisms in both acidic and alkaline environments, the abundance of protons (H⁺) in acidic media allows for a more straightforward pathway for H* adsorption, requiring relatively low activation energy. In contrast, alkaline conditions involve the dissociation of H_2_O molecules, making the Volmer step a kinetically limiting process due to the additional energy needed for H_2_O dissociation.

Building on this understanding of HER mechanisms, we investigated HER behavior using DFT by employing the computational hydrogen electrode (CHE) model and free energy diagram (FED) approach proposed by Nørskov et al. to accurately describe HER activity [12]. This model equates the paired energy of a proton and an electron to half the energy of a H_2_ molecule under standard conditions (applied potential (*U*) = 0.0 V, pH = 0, *p* = 1 atm, *T* = 298 K), expressed as:

4 $$G(H^{ + } + {e}^{ - } ) = { G}\left( {1/2{H}_{2} } \right),\Delta {G}^{0} = 0{eV}$$

The CHE model considers the thermodynamics of the reaction and links it to the equilibrium potential. Particularly, when hydrogen is adsorbed onto the surface, the Gibbs free energy of adsorption ($\Delta {G}_{{H}^{*}}$) is given by:

5 $$\Delta {G}_{{{H}^{*} }} = { }\Delta {E}_{{{H}^{*} }} + \Delta {ZPE} - {T}\Delta {S}$$

where, $\Delta {E}_{{H}^{*}}$ is the H*-adsorption energy, and $\Delta ZPE$ and $\Delta S$ are the changes in zero-point energy and entropy associated with hydrogen adsorption, respectively, and $T$ is the temperature [26, 27].

Figure 3b shows that, the Volmer step involving water molecules is described using the following equation when the HER occurs under alkaline conditions [25]:

6 $$Volmer\ step:H_{2} O + e^{ - } + * \to H^{*} + OH^{ - }$$

A free-energy relationship based on the CHE model is derived to determine alkaline HER activity initiated by water, which can be formulated as follows under standard conditions:

7 $${\text{H}}_{2} {\text{O}}\left( {\text{l}} \right) + {\text{e}}^{ - } \to { }0.5{\text{H}}_{2} + {\text{OH}}^{ - } { }\left( {E^{0} = - 0.828{\text{ V}}} \right)$$

where ${E}^{0}$ is the standard reduction potential at *T* = 298 K. The chemical potential in this reaction is given by:

8 $$\mu_{{e^{ - } }} - \mu_{{OH^{ - } }} = 0.5\mu_{{H_{2} \left( g \right)}} - \mu_{{H_{2} O\left( l \right)}}$$

Here ${\mu }_{{H}_{2}O\left(l\right)}$ and ${\mu }_{{H}_{2}\left(g\right)}$ are calculated using the approximations of Nørskov et al., where ${\mu }_{{H}_{2}O\left(l\right)}$ is equal to ${\mu }_{{H}_{2}O\left(g\right)}$ at *T* = 298 K and 0.035 bar [28]. Therefore, Eq. 8 can be written as:

9 $$\mu_{{e^{ - } }} - \mu_{{OH^{ - } }} = 0.5\mu_{{H_{2} \left( g \right)}} - \mu_{{H_{2} O\left( g \right)}}$$

HER activity under alkaline conditions is then evaluated by establishing the FED for the following reaction equations:

10 $$\Delta G_{1} = G_{{H_{2} O^{*}}} - G_{*} - \mu_{{H_{2} O\left( g \right)}}$$

11 $$\Delta G_{2} = G_{{H^{*} /OH^{*} }} - G_{{H_{2} O^{*}}}$$

12 $$\Delta G_{3} = G_{{H^{*} }} + \mu_{{OH^{ - } }} - G_{{H^{*} /OH^{*} }} - \mu_{{e^{ - } }}$$

The binding free energies (${\Delta G}_{{H}_{2}{O}^{*}}$, ${\Delta G}_{{H}^{*}/O{H}^{*}}$, and ${\Delta G}_{{H}^{*}}$) to the Volmer step during the alkaline HER is calculated by determining $\Delta {G}_{1}$, $\Delta {G}_{2}$, and $\Delta {G}_{3}$ [29, 30].

### Evaluating OER activity under acidic and alkaline conditions: A DFT perspective

With the aim of optimizing OER activity in acidic and alkaline environments, employing a DFT perspective enables detailed analyses using the CHE and FED approaches, as proposed by the Nørskov group. The thermodynamic stabilities of intermediates, which serve as critical descriptors of catalytic performance, are key aspects when investigating the OER [31–33]. The OER mechanism unfolds through a four-electron, four-step process under acidic conditions, as described by the following reactions (Eqs. 13-16, Fig. 3c):

13 $$*+{\text{H}}_{2}\text{O}\left(\text{l}\right)\to \text{ O}{\text{H}}^{*}+{(\text{H}}^{+}+{\text{e}}^{-})$$

14 $${\text{OH}}^{*}\to {\text{O}}^{*}+{(\text{H}}^{+}+{\text{e}}^{-})$$

15 $${\text{O}}^{*}+{\text{H}}_{2}\text{O}(\text{l})\to {\text{OOH}}^{*}+{(\text{H}}^{+}+{\text{e}}^{-})$$

16 $${\text{OOH}}^{*}\to * + {\text{O}}_{2}+{(\text{H}}^{+}+{\text{e}}^{-})$$

where, * denotes the active site for the OER, and OH*, O*, and OOH* represent intermediates adsorbed on the catalyst surface. The Gibbs free energy of each step in acidic media is calculated as follows Eqs. 17-20:

17 $$\Delta {G}_{1}={G}_{O{H}^{*}}+{G(H}^{+}+{e}^{-})-({\mu }_{{H}_{2}O(l)}+{G}_{*})$$

18 $$\Delta {G}_{2}={G}_{{O}^{*}}+{G(H}^{+}+{e}^{-})-({G}_{O{H}^{*}})$$

19 $$\Delta {G}_{3}={G}_{OO{H}^{*}}+{G(H}^{+}+{e}^{-})-({\mu }_{{H}_{2}O(l)}+{G}_{{O}^{*}})$$

20 $$\Delta {G}_{4}={G}_{*}+{\mu }_{{O}_{2}}+{G(H}^{+}+{e}^{-})-({G}_{O{OH}^{*}})$$

These $\Delta {G}_{n}$ values can be calculated using the chemical potentials of a H_2_(g) half as a proton/electron couple, liquid water, and oxygen molecules based on the following relationship: $2{\text{H}}_{2}\text{O}\to {\text{O}}_{2}+2{\text{H}}_{2}\text{ at }4.92\text{ V}$ and the free energy of each intermediate (*G*_OH*_, *G*_O*_, and *G*_OOH*_) on the active surface site (*). The degree of the OER potential-determining step (${G}^{OER}$) in the four-electron reaction steps, which is a crucial electrocatalytic-activity parameter, can be determined from the calculated $\Delta {G}_{n}$ values; it corresponds to the specific reaction point with the largest $\Delta {G}_{n}$ in the elementary reaction steps of the OER, i.e., the concluding step that achieves a downhill reaction in the FED with increasing potential:

21 $${G}^{OER}= \text{max}[\Delta G_{1}, \Delta G_{2}, \Delta G_{3}, \Delta G_{4}]$$

After identifying the largest $\Delta {G}_{n}$ value, which determines the bottleneck step in the OER, the theoretical overpotential (${\eta }^{OER}$) under acidic conditions is finally determined using the following equation:

22 $${\eta }^{OER}={(G}^{OER}/e)-x\text{V}, x=1.23\text{ V}$$

The calculated ${\eta }^{OER}$ is the required potential to overcome the bottleneck step in the acidic OER.

On the other hand, the alkaline OER is described by the following reactions (Eqs. 23-26, Fig. 3d):

23 $${\text{OH}}^{-}+ * \to \text{ O}{\text{H}}^{*}+{\text{e}}^{-}$$

24 $${\text{OH}}^{*}+{\text{OH}}^{-}\to {\text{O}}^{*}+{\text{H}}_{2}\text{O}(\text{l})+{\text{e}}^{-}$$

25 $${\text{O}}^{*}+{\text{OH}}^{-} \to \text{ OO}{\text{H}}^{*}+{\text{e}}^{-}$$

26 $${\text{OOH}}^{*}+{\text{OH}}^{-} \to {\text{O}}_{2}(\text{g})+{\text{H}}_{2}\text{O}(\text{l})+{\text{e}}^{-}$$

The $\Delta {G}_{n}$ value of each OER step in an alkaline medium can be expressed as follows (Eqs. 27-30):

27 $$\Delta {G}_{1}={G}_{O{H}^{*}}+{\mu }_{{e}^{-}}-({\mu }_{{OH}^{-}}+{G}_{*})$$

28 $$\Delta {G}_{2}={G}_{{O}^{*}}+{\mu }_{{H}_{2}O(l)}+{\mu }_{{e}^{-}}-({{G}_{OH*}+G}_{*})$$

29 $$\Delta {G}_{3}={G}_{{OOH}^{*}}+{\mu }_{{e}^{-}}-({G}_{{O}^{*}}+{\mu }_{O{H}^{-}})$$

30 $$\Delta {G}_{4}={G}_{*}+{\mu }_{{O}_{2}}+{\mu }_{{H}_{2}O(l)}+{\mu }_{{e}^{-}}-({G}_{{OOH}^{*}}+{\mu }_{O{H}^{-}})$$

These $\Delta {G}_{n}$ values are calculated using the chemical potentials of hydroxide, an electron, liquid water, and O_2_(g) (${\mu }_{{OH}^{-}}$, ${\mu }_{{e}^{-}}$, ${\mu }_{{H}_{2}O(l)}$, and ${\mu }_{{O}_{2}}$) and the free energy of each intermediate (*G*_OH*_, *G*_O*_, and *G*_OOH*_), which leads to ${\eta }^{OER}$ under alkaline conditions described by the following equation:

31 $${\eta }^{OER}={(G}^{OER}/e)-x\text{V}, x=0.402\text{ V}$$

Based on the perspective presented above, ${\eta }^{OER}$ can be determined for each catalytic environment.

The well-defined FED approach, which is grounded in the CHE model, enables the thermodynamic behavior of electrocatalyst reactions in both acidic and alkaline environments to be systematically evaluated. This approach is illustrated in Fig. 3e, where FED analysis of the HER on the Ni_3_P(111) surface reveals that H* at the phosphorus (P) site is more conducive for H_2_ evolution than at the nickel (Ni) site [34]. $\Delta {G}_{{H}^{*}}$ values close to zero suggest that H* is an optimal site for efficient H_2_ evolution, thereby minimizing risks of hydrogen poisoning from excessively strong binding or lower HER reactivity due to overly weak binding.

In alkaline media, where H_2_O molecules serve as the primary proton source, the HER pathway fundamentally differs from that in acidic conditions. The dissociation of H_2_O into H* and OH* introduces a kinetic barrier, emphasizing the critical role of catalyst design in lowering this barrier and enhancing HER kinetics under such conditions. Notably, in the case of Ru_2_P(010), the dissociation of H_2_O* into H*/OH* occurs spontaneously, as shown in the FED analysis in Fig. 3f [35], demonstrating its suitability for facilitating the Volmer step. In contrast, the Ir_2_P(111) structure faces an energetic uphill barrier despite exhibiting ideal $\Delta {G}_{{H}^{*}}$ values for H_2_ evolution. This analysis shows that Ru_2_P(010) and Ir_2_P(111) affect H_2_O activation and dissociation differently, which significantly impacts their overall HER efficiencies.

The same intermediate stages involving OH*, O*, and OOH* are followed in the OER under both acidic and alkaline conditions; however, the behavior of the reaction depends significantly on the catalytic environment. This variance is ascribable to the different initiating species: water molecules under acidic conditions and hydroxide ions under alkaline conditions. For instance, Fig. 3g illustrates OER behavior under acidic conditions on an armchair nitrogen-doped graphene structure at various potentials (0, 1.23, and 1.635 V) that correspond to zero, equilibrium, and limiting potentials for the acidic OER, respectively [36]. Figure 3h depicts OER behavior under alkaline conditions on the carbon sites of a Mn/Fe-doped hexaiminobenzene metal–organic framework (MOF) at potentials of 0, 0.402, and 0.77 V, which represent the zero, equilibrium, and limiting potentials of the alkaline OER, respectively [37]. These diagrams show activities at specific catalytic sites across a range of applied potentials, which highlights the nuanced interplay between catalyst structure, environment, and electrocatalytic activity. Building on these observations, DFT methods play a crucial role in providing detailed insights into the thermodynamic properties of HER and OER mechanisms. While techniques like the nudged elastic band (NEB) are available for exploring kinetic aspects, this review primarily focuses on thermodynamic descriptors, particularly Gibbs free energy changes, as the primary framework for understanding and assessing HER and OER activity.

## DFT-simulation-based materials perspectives

### Key catalyst materials for water-splitting reactions

**Fig. 4:**
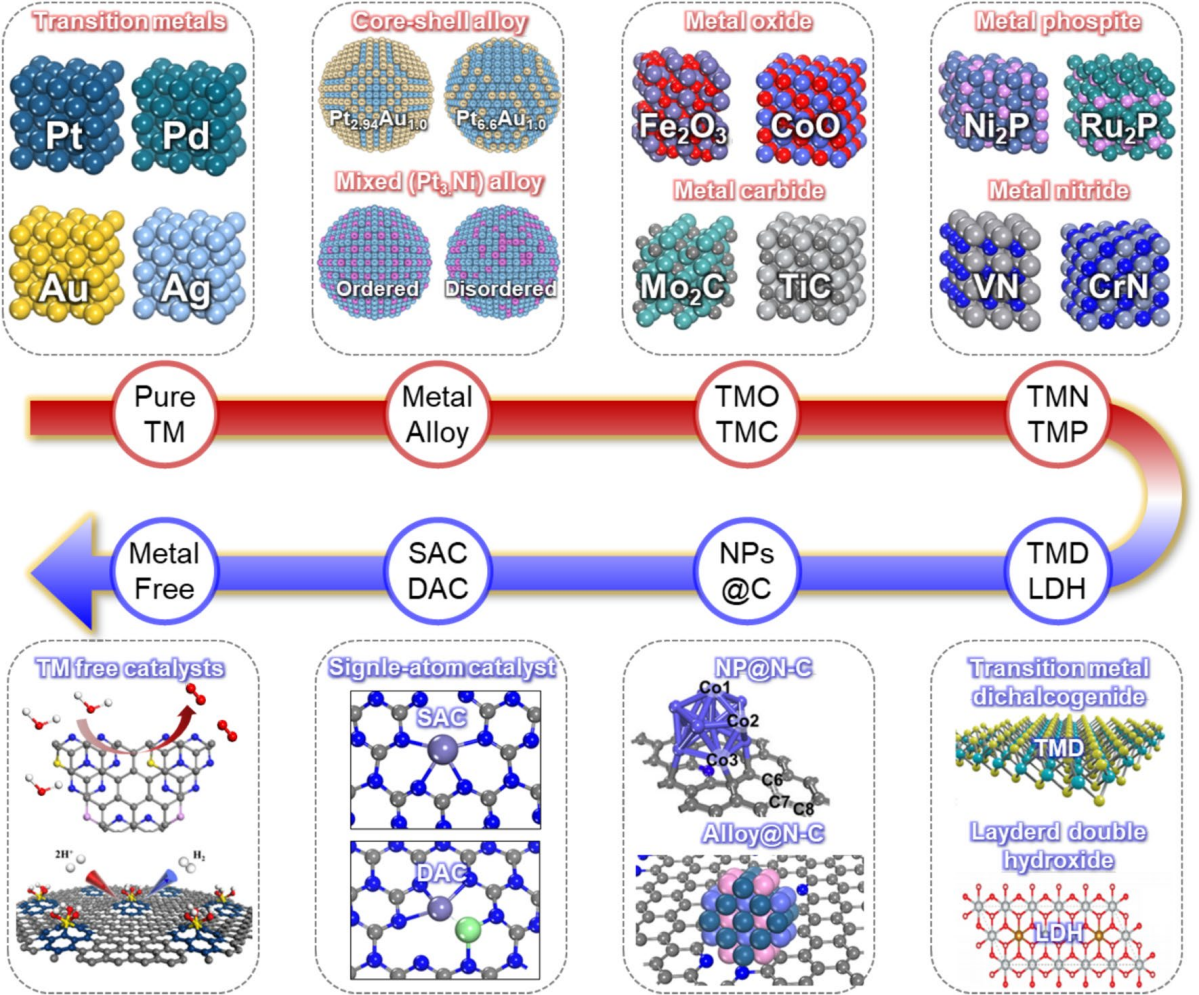
Schematic depicting the evolution and diversity of water-splitting catalysts, categorized by composition and structural complexity. The progression from pure transition metals (e.g., Pt, Pd, Au, and Ag) to more advanced materials, such as core–shell alloys, metal oxides, carbides, phosphides, and nitrides, illustrates the shift to more efficient and cost-effective electrocatalysts. To reduce metal content, the figure also highlights the development of advanced 2D materials (e.g., transition-metal dichalcogenides, layered double hydroxides, referred to as TMDs and LDHs, respectively), using N-doped carbon supports for anchoring nanoparticles (NPs), and transition-metal-free catalysts (e.g., non-TM catalysts, single-atom, or double-atom catalysts, referred to as SACs or DACs). These materials represent promising alternatives to traditional metal-based systems, and exhibit potential for enhancing catalytic performance in both the HER and OER. (Figure panels are reproduced with permission [29, 59, 63, 66, 76, 86])

The evolution of catalyst materials for use in water-splitting applications is a testament to the dynamic interplay between experimental insight and theoretical advancements. As shown in Fig. 4, the journey began with pristine TMs that formed the cornerstones of the initial water-splitting systems. Fundamentally, these first-generation electrocatalysts are limited by the high costs and low activities of precious TMs [25, 38, 39]. To circumvent these constraints, research pivoted toward metal-alloy systems, notably core–shell and mixed-alloy structures. These alloys are favored for efficient metal utilization and enhanced catalytic performance, although challenges such as precisely controlling their sub-5 nm sizes and alloy mixing ratios persist [40–46].

Subsequent advancements led to the exploration of metal-based compounds, such as oxides (TMOs), carbides (TMCs), phosphides (TMPs), and nitrides (TMNs). These materials, with their diverse compositions and tunable metal ratios, contributed significantly to practical catalyst durability. Notably, the ability to control morphologies on the nanoscale further amplified catalytic performance [47–57]. Despite these advances, issues such as long-term stability and efficiency relative to metal usage persist because catalytic reactions predominantly occur on surfaces.

Metal-containing 2D structures have been studied with the aim of increasing metal efficiency and improving catalyst stability, with representative examples including transition-metal dichalcogenides (TMDs) and layered double hydroxides (LDHs). TMDs, which are characterized by their MX_2_ chemical formulas (where M is a transition metal such as Mo, W, or Ti, and X is a chalcogen such as S, Se, or Te), exhibit superior HER/OER catalytic performance owing to their unique thin layers and electronic structures [58–61]. LDHs with the general ${\left[{M}_{1-x}^{2+}{M}_{x}^{3+}{\left(OH\right)}_{2}\right]}^{x+}{\left[{A}^{n-}\right]}_{x/n}\cdot m{H}_{2}O$ formula offer versatile cation (${M}^{2+}/{M}^{3+}$) and anion (${A}^{n-}$) combinations that result in a wide range of chemical properties, which led to their widespread application, particularly as HER/OER electrocatalysts [62–65]. Furthermore, significant effort has been directed toward using N-doped carbon as a support material to combat catalyst degradation. N-doped carbon structures, such as pyridinic, pyrrolic, graphitic, and pyrazole N sites, as well as N-doped reduced graphene oxide (NrGO), provide robust anchoring sites that prevent metal-particle agglomeration during catalytic reactions, thereby preserving their activities [66–69]. Additionally, a plethora of 2D substrates, such as g-C_3_N_4_, h-BN, C_2_N, and MXenes, have been investigated with the aim of further enhancing catalytic stability and activity [70–74].

Single-atom catalysts (SACs) have also attracted considerable interest for maximizing metal-site efficiency while minimizing metal content. SACs have exhibited superior catalytic activities when supported by various materials, including N-doped carbon, g-C_3_N_4_, oxides, and carbides, as evidenced through both experimental and theoretical studies. In addition [75–81], double-atom catalysts (DACs) on substrates such as NrGO, g-C_3_N_4_, and phthalocyanines have leveraged metal-atom synergy to enhance catalytic activity [82–85].

Finally, the development of TM-free catalysts has emerged as a critical frontier. Doping carbon-based catalysts with non-metal elements, such as phosphorus, sulfur, and nitrogen, offers a promising route, with these TM-free catalysts delivering catalytic performance comparable to that of their metal-based counterparts. These catalysts not only exhibit enhanced catalytic activities and stabilities but also provide significant benefits in terms of cost-effectiveness and environmental sustainability [29, 86–89].

### Theoretical strategies for enhancing HER and OER performance

**Fig. 5:**
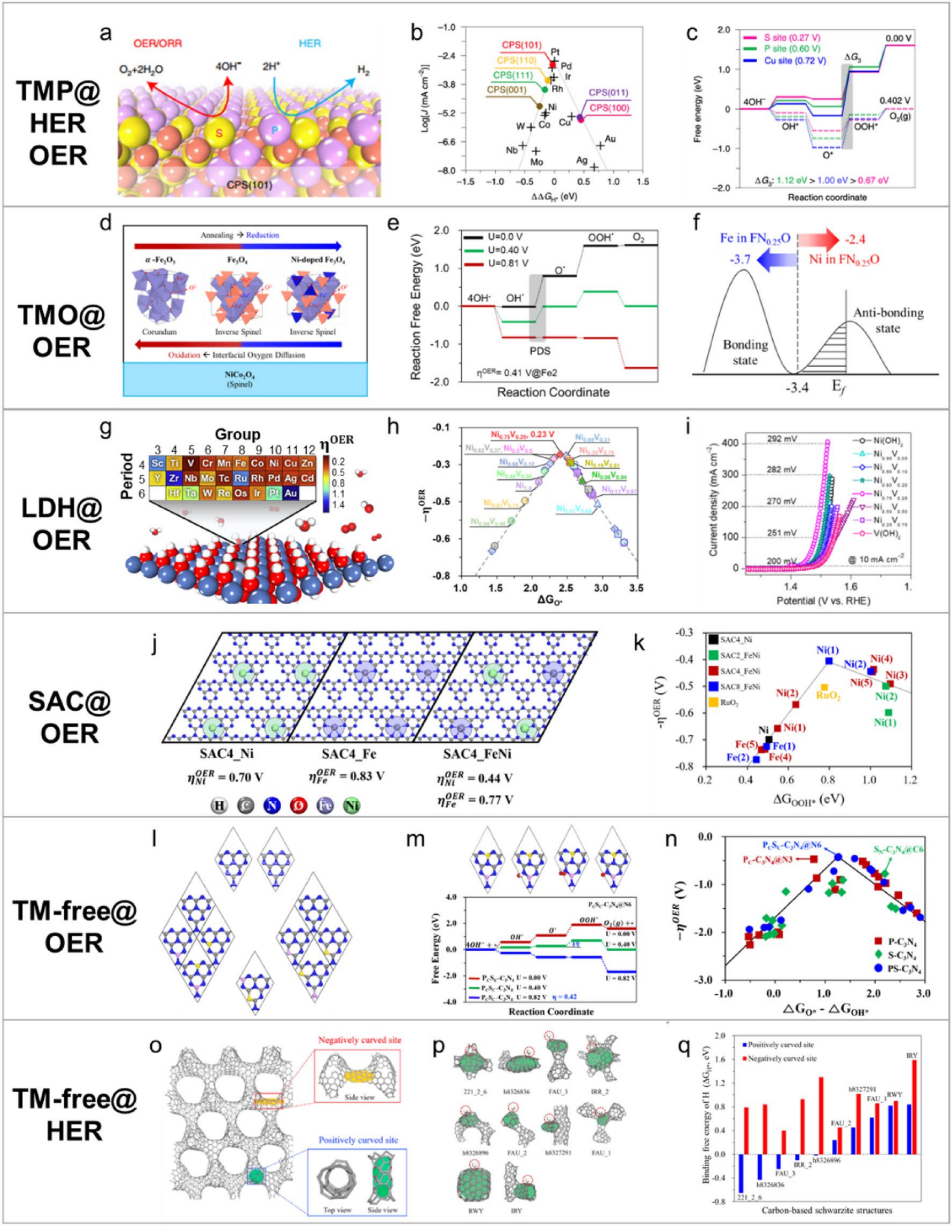
Representative research into HER and OER catalysis, showing various approaches and material systems for enhancing catalytic performance. **a–c** Multifunctional catalytic properties of copper phosphosulfide (CPS(101)) as a TMP catalyst: **a** schematic image, **b** volcano plot for the HER, and **c** FED for the OER. **d–f** Catalytic properties of inverse spinel Fe_3_O_4_@NiCo_2_O_4_ (rFNCO) as a TMO catalyst for the OER: **d** structure-design process, **e** FED for the OER, and **f** schematic showing the *d*-band centers of Fe and Ni in the catalyst. **g–i**: Screening $N{i}_{1-x}T{M}_{x}$ LDH candidates ($0\le x\le 1$, TM = V and Fe) for the OER: **g** schematic image, **h** volcano plots for the OER using potential catalysts, and **i** experimental linear sweep voltammetry (LSV) curves that validate theoretical catalytic performance. **j–k**: Superior catalytic performance of Fe/Ni bimetallic active sites on TM@g-C_3_N_4_ for the OER as a single-atom catalyst: **j** overpotential as a function of metal composition and **k** volcano plots for the OER based on possible SACs. **l–n**: Investigating TM-free electrocatalysts, focusing on B,S-doped N–C structures for the OER: **l** various non-metal doping configurations, **m** an FED for the OER, and **n** volcano plot for potential doped catalysts. **o–q**: HER activities of carbon schwarzites with unique geometric properties: **o** positive/negative curvatures, **p** representative schwarzite structures, and **q** relationship between curvature and H*-binding free energy. (Figure panels are reproduced with permission form [29, 76, 90–93])

The effectiveness of DFT simulations for analyzing various catalysts is demonstrated by examining six distinct cases involving TMP-, TMO-, LDH-, SAC-, and TM-free catalysts. One such example (shown in Fig. 5a–c) involves the research reported by Shinde et al. on the multifunctional copper phosphosulfide (CPS(101)) catalyst, which is used for both the HER and OER [90]. Their findings highlighted synergistic interactions between copper, phosphorus, and sulfur on the CPS(101) surface that significantly enhance catalytic performance. DFT simulations revealed how P and S are spatially separated on the surface, with P designated as the proton acceptor site for the HER and S as the O-species acceptor site for the OER. This separation results in a Gibbs free energy for hydrogen adsorption ($\Delta {G}_{{H}^{*}}^{DFT})$ of $-0.05 eV ({ \eta }_{OER}^{DFT}=0.27 V).$ DFT analysis also revealed that the experimental activities are closely correlated with the electronic structure, particularly for different antibonding behavior at the P and S active sites. This insight underscores the roles that electronic interactions play in dictating catalytic activity and demonstrates the utility of DFT in uncovering complex surface phenomena.

Jo et al. investigated the catalytic properties of the inverse spinel Fe_3_O_4_@NiCo_2_O_4_ (rFNCO) TMO structure (Fig. 5d–f), focusing on the borohydride-based reduction process, which enhances OER performance [91]. From a DFT perspective, the ${\left[F{e}_{0.75}N{i}_{0.25}\right]}^{{T}_{d}}F{e}_{2}^{{O}_{h}}{O}_{4}$ structure, where Ni diffuses from NiCo_2_O_4_ into Fe_3_O_4_, exhibits atomic Fe/Ni synergy that improves O* binding at key intermediates and enhances OER activity as a consequence (${\eta }_{OER}^{DFT}=0.41 V$). This improvement is attributable to the optimal shifting of *d*-band centers at active sites.

As a promising LDH-based catalyst, Chavan et al. employed DFT simulations to screen various metal combinations in $N{i}_{1-x}T{M}_{x}$ LDHs ($0\le x\le 1$, TM = V and Fe) and identified Ni_0.75_V_0.25_ LDH as the most promising catalyst for the OER owing to its highest number of active O_VNiNi_ sites and lowest hydrogen desorption energy (HDE), with ${\eta }_{OER}^{DFT}=0.23 V$ (Fig. 5g–i) [92]. The optimized catalyst was then synthesized and experimentally examined, demonstrating an ultralow overpotential and excellent long-term stability. This study highlights synergy between theoretical predictions and experimental validation, thereby providing a robust approach for designing electrocatalysts.

Shin et al. investigated the superior catalytic performance of Fe/Ni bimetallic active sites on TM@g-C_3_N_4_ as single-atom catalysts (SACs) for the OER (Fig. 5j–k) [76]. In this study, the DFT approach revealed that the experimentally observed catalytic activity is driven by a structural corrugation effect induced by the specific arrangement of Fe and Ni atoms and their interactions in both 2D (${\eta }_{OER}^{DFT}=0.41 V$) and 3D (${\eta }_{OER}^{DFT}=0.45 V$) TM@g-C_3_N_4_. These findings underscore the significant role that each TM site and configuration play in tuning both the geometric and electronic structures, leading to superior catalytic efficiency compared with those of SACs containing only Ni or Fe.

Given the focus on TM-free catalysts, Lee et al. subsequently conducted a comprehensive study on TM-free electrocatalysts for the OER in alkaline media, focusing on the structure–catalytic-activity relationship (Fig. 5i–n) [29]. Using DFT simulations, they systematically investigated the electronic and geometric effects of heteroatom doping on the g-C_3_N_4_ structure, and identified P and S co-doped g-C_3_N_4_ as the most promising catalyst owing to geometric and electronic synergies leading to enhanced OER performance (${\eta }_{OER}^{DFT}=0.42 V$). In addition, Seok et al. comprehensively investigated the HER activities of carbon schwarzites with unique structures and large surface areas using DFT simulations, focusing on their geometric and electronic properties (Fig. 5o–q) [93]. An evaluation of 64 different schwarzite structures identified the h8326896 structure as the most promising candidate owing to its close-to-ideal optimal hydrogen-binding free energy ($\Delta {G}_{{H}^{*}}=-0.02 eV$). This study revealed that the superior catalytic activity is driven by the positive curvatures of carbon sites and favorable *p*-orbital characteristics, which enhance hydrogen adsorption and desorption. These findings underscore the potential of h8326896 as a cost-effective and Earth-abundant alternative to traditional TM-based catalysts for the HER and OER.

## Binding-energy correlations and electronic-structure analysis

### Scaling relationships and volcano plots of catalytic-activity trends

**Fig. 6:**
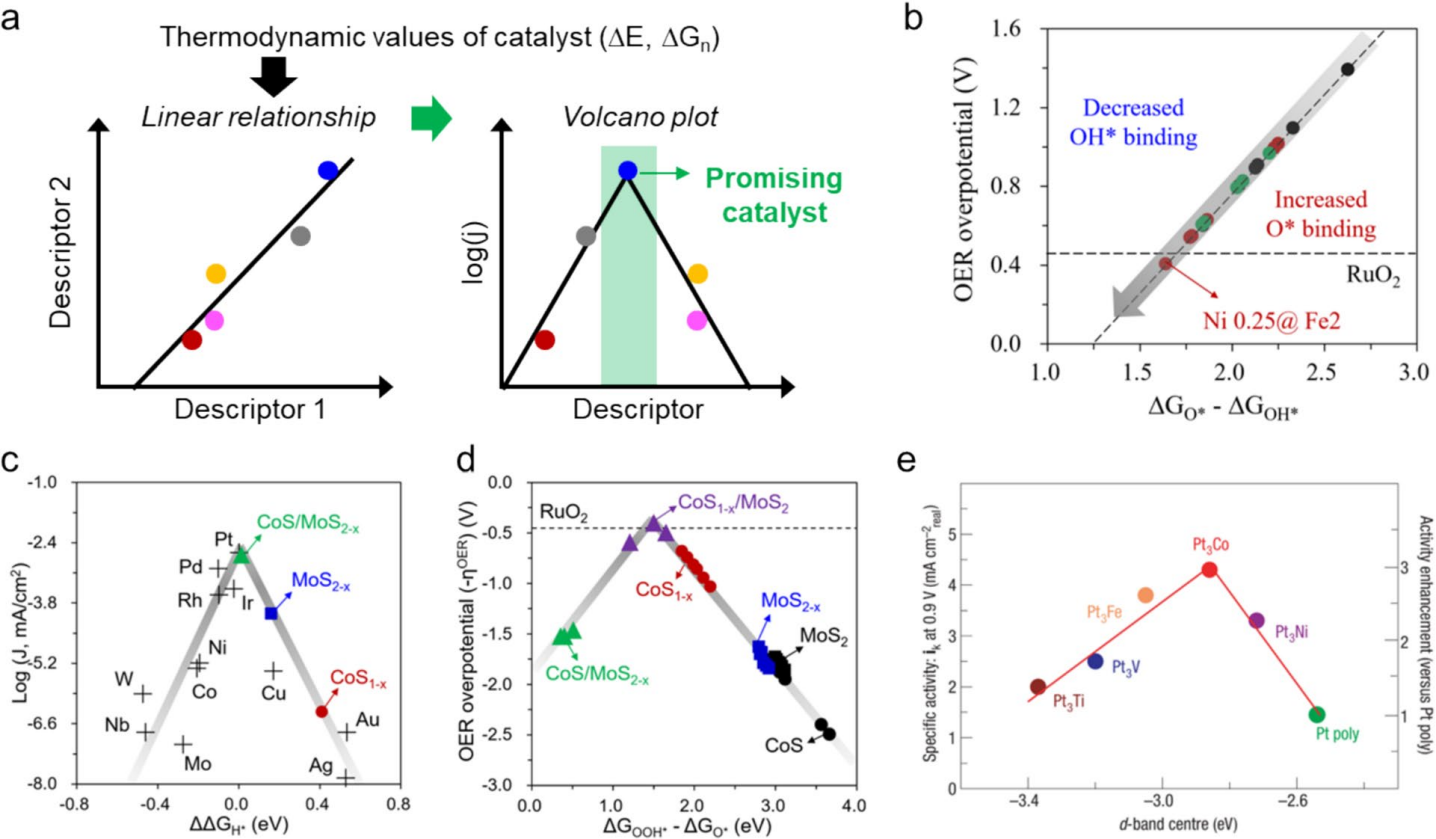
**a **Schematic showing the linear scaling relationship between thermodynamic descriptors and volcano plots, highlighting promising catalyst candidates. **b **Scaling relationship between OER overpotential and ΔG_O*_ - ΔG_OH*_ for all metal active sites. Black, green, and red circles represent the metal sites in the Fe_3_O_4_, FeNi_0.25_O, and FeNi_0.375_O structures, respectively, and show how different compositions influence OER activity. **c **Volcano plot of experimental log(j) against theoretically calculated ΔΔG_H*_ for the HER over CoS/MoS_2-X_, MoS_2-X_, and CoS_1-X_ with various metals included (Pt, Pd, Rh, Ir, Ni, Co, W, Nb, Mo, Cu, Ag, and Au). **d **OER volcano plot showing the relationship between η^OER^ and ΔG_OOH*_ - ΔG_O*_ for designed catalyst structures. **e **Relationship between experimentally measured specific ORR activities on Pt_3_M surfaces in 0.1 M HClO_4_ at 333 K and *d*-band center positions. (Figure panels are reproduced with permission [91, 98, 99])

The process of designing theoretical catalyst models and examining their activities is intricate, multifaceted, and focuses on deciphering the origins of catalytic performance and identifying promising catalyst candidates. Central to this endeavor is the use of DFT calculations to establish linear scaling relationships among key thermodynamic descriptors, including adsorption energies, reaction Gibbs free energies, and limiting potentials. These relationships are crucial for deepening our understanding of the structure–property correlations that operate in these catalysts, as illustrated in Fig. 6a. Indeed, they provide essential insight for identifying and overcoming specific bottlenecks in catalyst research. A critical step in this analytical process involves identifying the most influential thermodynamic descriptor that affects catalytic activity, which is instrumental when constructing volcano plots that graphically represent the Sabatier principle. According to this principle, the optimal catalyst is characterized by moderate binding affinities for reaction intermediates. Such affinities ensure that the catalyst does not bind too strongly or too weakly to the intermediates, thereby ensuring optimal catalytic performance. Balancing this interaction is the key to maximizing efficiency and is pivotal for guiding the development of new catalysts capable of meeting rigorous performance criteria [94–97].

With the aim of enhancing OER activity, Jo et al. synthesized Fe_3_O_4_@NiCo_2_O_4_ (rFNCO) using a borohydride-based reduction process and explored its catalytic activity [91]. These researchers constructed ${\left[F{e}_{1-x}N{i}_{x}\right]}^{{T}_{d}}F{e}_{2}^{{O}_{h}}{O}_{4} (x=0, 0.25, {\text{and }} 0.375)$ (rFNCO) structures by incorporating Ni atoms into Fe_3_O_4_ via diffusion from NiCo_2_O_4_, focusing on atomic synergy between Fe and Ni. The rFNCOs exhibited calculated ${\eta }^{OER}$ values that are linearly related to Gibbs free energy differences for O* and OH* binding (i.e. $\Delta {G}_{{O}^{*}}-\Delta {G}_{O{H}^{*}}$) (Fig. 6b). Stronger O* binding and weaker OH* binding led to lower ${\eta }^{OER}$ values, with the lowest value of $\Delta {G}_{{O}^{*}}-\Delta {G}_{O{H}^{*}}$ achieved when optimal amounts of Ni atoms were incorporated, particularly at Fe–Ni bidentate sites for O* adsorption, which indicates that OER activity is significantly affected by the O*-binding affinity over binding to other intermediates, based on scaling relationships in the rFNCO catalysts.

As examples of well-defined volcano plots, Ahmed et al. explored highly efficient and robust water electrolysis by enhancing HER and OER activities. These researchers synthesized CoS/MoS_2-X_ and CoS_1-X_/MoS_2_ as hybrid heterostructures of CoS and MoS_2_ nanoparticles, with S-defects incorporated on each phase to maximize the known catalytic properties of both CoS (OER) and MoS_2_ (HER) [98]. The volcano plot depicted in Fig. 6c shows the relationship between the experimental current density (log(j)) and $\Delta \Delta{G}_{{H}^{*}}$ value ($\Delta \Delta {G}_{{H}^{*}} = \Delta {G}_{{H}^{*}}-\Delta {G}_{Pt-{H}^{*}})$. Using these plots, the authors emphasized that the hybridized CoS/MoS_2-X_ exhibited Pt-like HER activity, with the highest log(j) and moderate H* binding affinity, thereby outperforming the MoS_2-X_ and CoS_1-X_ structures. Similarly, OER performance was also investigated, as shown in Fig. 6d. The volcano plot of the relationship between ${\eta }^{OER}$ and $\Delta {G}_{{OOH}^{*}}-\Delta {G}_{{O}^{*}}$ showed that CoS_1-X_ is more effective in lowering ${\eta }^{OER}$ compared to MoS_2-X_. Importantly, the hybridized CoS_1-X_/MoS_2_ exhibited near-optimal binding energies for these intermediates, thereby positioning the hybrid system close to the peak of the volcano plot where maximum OER activity is observed while outperforming RuO_2_.

In addition to binding affinity, the electronic properties of a catalyst, such as orbital band centers, *e*_*g*_ state electrons, and other electronic features, are critical determinants of catalytic performance [100, 101]. These properties are effectively visualized and analyzed using volcano plots, which provide powerful graphical representations how electronic structure influences catalytic activity. A notable example includes the use of volcano plots based on *d*-band center values for Pt_3_M catalysts under acidic oxygen evolution reaction (ORR) conditions, as shown in Fig. 6e. This methodological approach underscores the pivotal role played by the optimal *d*-band center in catalysis, which suggests that moderately filled antibonding states below the Fermi level are ideal for enhancing catalytic activity. This balance facilitates effective bond formation and cleavage during the reaction, which is essential for efficient catalysis. Our understanding of the fundamental aspects of catalytic behavior is significantly enhanced by integrating the electronic descriptors derived from DFT simulations. This insight not only illuminates the key factors that govern reaction mechanisms, but also drives the development of more sophisticated and efficient electrocatalysts. Such detailed analyses enable researchers to tailor catalyst properties in order to optimize performance and address specific electrochemical challenges.

### Charge density and its impact on catalytic behavior

Understanding the overall catalytic behavior in an electrochemical reaction necessitates examining the charge states within the catalyst structure, as proton/electron interactions are profoundly influenced by these charge states. To this end, analyzing charge-density distributions is fundamental for exploring how electrocatalysts facilitate crucial reaction steps and devising strategies to augment catalytic activity. Common techniques employed for this purpose include Bader charge analysis, Mulliken population analysis, and Löwdin charge calculations; these methods are instrumental in numerically quantifying electron gain or loss within the catalyst structure, and offer crucial insight into charge-state redistribution during reactions such as the HER and OER. This analysis helps to fine-tune catalyst designs to enhance performance [102–104].

**Fig. 7:**
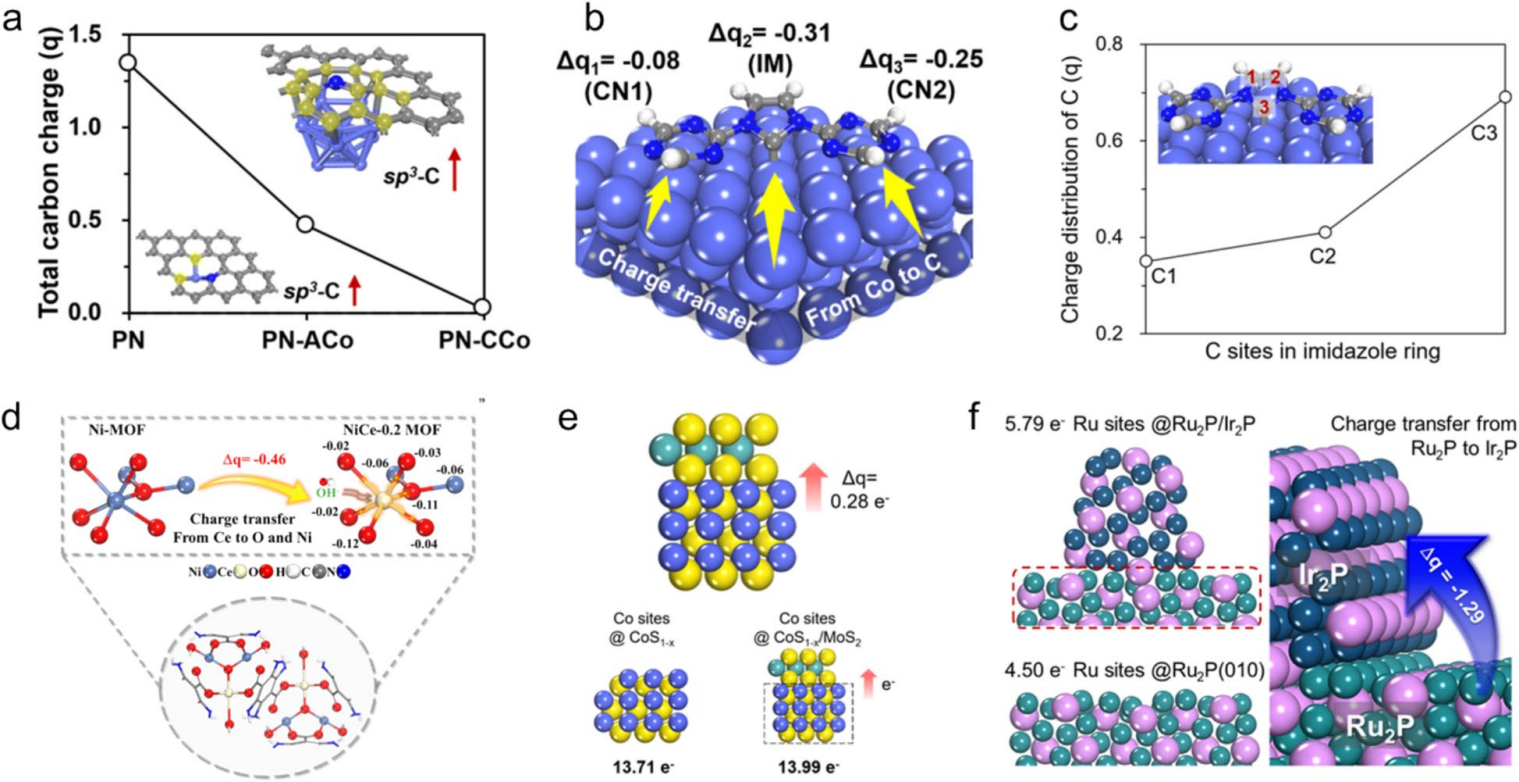
**a** Structural-deformation-induced *sp*^*3*^-C sites and total charge variations (q) of the carbons on the carbon sheet doped with only pyridinic N (PN) and additional atomic Co (PN-ACo) or clustered Co (PN-CCo). The yellow circles highlight the deformed area and *sp*^*3*^-C sites. **b** Schematic depicting electronic charge transfer (Δq) from the Co surface to the C sites of the imidazole (IM) and triazine (TR) moieties, and **c** corresponding electronic charge distribution at the C sites of the imidazole group in the Co-nanoparticle-incorporated spherical imidazolium-rich COF-derived electrocatalyst (CoNP-s-IMCOF). **d** Illustrating Ce^3+^-induced charge transfer in the Ce-doped Ni-metal framework (MOF) structure. **e** Charge transfer in CoS_1-x_/MoS_2_ and **f** from Ru_2_P(010)/Ir_2_P(111) heterojunction. (Figure panels are reproduced with permission [35, 66, 98, 105, 107])

Moreover, charge distribution at the active sites is directly shaped by the geometric structure of the catalyst. Therefore, investigating how charge states are altered following structural modifications, such as heteroatom doping, structural deformation, and heterostructure engineering, is vital to ensure a deeper understanding of catalytic behavior. For instance, Park et al. developed pyridinic-nitrogen (PN) doped carbon sheets with incorporated atomic cobalt (PN-ACo) or cobalt clusters (PN-CCo) to induce structural deformation and create *sp*^*3*^-hybridized carbon sites that enhance OER performance, as depicted in Fig. 7a [66]. Charge-state analysis revealed that PN-CCo is almost charge-neutral, in contrast to the positively charged states that exist in the PN and PN-ACo structures. The PN-CCo structure, with its high concentration of *sp*^*3*^-hybridized carbon sites, facilitates electron accumulation at these sites, thereby promoting more efficient charge transfer. This capability significantly contributes to the superior OER activity observed for the PN-CCo catalyst owing to its enhanced charge-transfer capacity. Such insight is invaluable for the development of more effective and efficient electrocatalysts by optimizing electronic interactions at active sites.

Ju et al. conducted an insightful study into spherical imidazolium-rich covalent organic frameworks of cobalt nanoparticles (CoNP-s-IMCOFs) to assess their viabilities as OER catalysts [105, 106]. These researchers specifically investigated the charge states of the imidazole moiety (IM) within the IMCOF structure and analyzed how these states are influenced by the presence of Co nanoparticles, and discovered that the IM moieties accepted substantial electronic charge from the CoNPs, indeed, more so than triazine moieties (TR1 and TR2), as shown in Fig. 7b. Further assessments revealed that certain carbon sites within the IM moiety are particularly effective OER active sites owing to their superior electron-accepting capabilities, as shown in Fig. 7c; such significant electron acceptance fosters a reductive environment at the carbon that enhances catalytic performance in the OER through dynamic interactions involving the IM moieties and CoNPs.

In another study that explored the effects of atomic doping on charge states, Shrestha et al. constructed site-selective disordered MIL88B(Ni) metal–organic frameworks (MOFs) through cerium (Ce) doping, resulting in the development of both Ni-MOF and Ce-doped NiCe-0.2 MOF structures for use in OER applications. Their study focused on charge-state variations induced by Ce doping, which revealed that Ce^3+^ ions in the NiCe-0.2 MOF catalyzed significant electron transfer between the Ce and Ni ions and the surrounding oxygen atoms, as depicted in Fig. 7d. The electron-deficient Ce^3+^ state proved critical because it facilitated interactions with electron-rich species such as OH⁻ during the OER process. Such interactions effectively lower the energy barrier for the OER, thereby improving the overall catalytic performance of the doped MOF structure. Such insight not only demonstrates the practical impacts of structural and electronic modifications on catalytic efficiency, but also underscores the importance of targeted atomic doping when optimizing electrocatalyst designs for enhanced electrochemical activity.

Heterostructure engineering plays a pivotal role in modulating charge-transfer dynamics within catalytic systems and can significantly affect performance. Notable examples include the hybrid CoS/MoS_2_-nanoparticle heterostructure, specifically the S-defected CoS_1-x_/MoS_2_ structure investigated by Ahmed et al. that demonstrated enhanced catalytic activity in each phase owing to strategic electron-transfer dynamics [98]. Electrons move from the CoS_1-x_ phase to the MoS_2_ phase in the CoS_1-x_/MoS_2_ system, which effectively donates electrons to the S sites of the MoS_2_, and creates varying charge states across the heterostructure, as illustrated in Fig. 7e. This electron redistribution results in an electron-rich MoS_2_ phase and an electron-deficient CoS phase, each offering distinct advantages: the MoS_2_ phase enhances reductive HER processes, whereas the CoS phase aids oxidative OER activities. Charge separation between these phases induces synergy that markedly improves the overall electrocatalytic efficiency.

Hong et al. explored the unique capabilities of Ir_2_P particles grown on a Ru_2_P surface structure (Ru_2_P/Ir_2_P) [35] and demonstrated exceptional alkaline HER activity. By delineating the roles played by each phase, namely Ru_2_P for water activation and dissociation and Ir_2_P for hydrogen production, these researchers uncovered significant synergy within the Ru_2_P/Ir_2_P heterostructure. In particular, substantial charge transfer from Ru_2_P to Ir_2_P at the interface between the two phases creates an electron-rich environment in the Ir_2_P phase that greatly enhances hydrogen production during the HER process. These case studies underscore the critical importance of analyzing charge in order to understand the electronic structures of catalytic materials. Insight into the electron-distribution and -transfer mechanisms are indispensable for designing efficient catalysts because they enable catalytic performance to be optimized across a range of reactions. This comprehensive understanding of charge dynamics facilitates the development of more effective electrocatalytic systems by tailoring the electronic environment to meet specific catalytic requirements.

### Electronic structure analysis using density of states (DOS)

Understanding the electronic structures of catalysts through the density of states (DOS) is fundamental for elucidating structure–property relationships in catalytic systems. The DOS provides the distribution of theoretically possible electronic states across a wide range of energy levels, thereby offering a comprehensive view of how electrons are organized within a material. This tool is critical for determining interactions between protons and electrons on the catalyst surface, and can be used to explain mechanisms associated with electron transfer and delineate the reactivities of electrons near the Fermi level (E_F_).

**Fig. 8:**
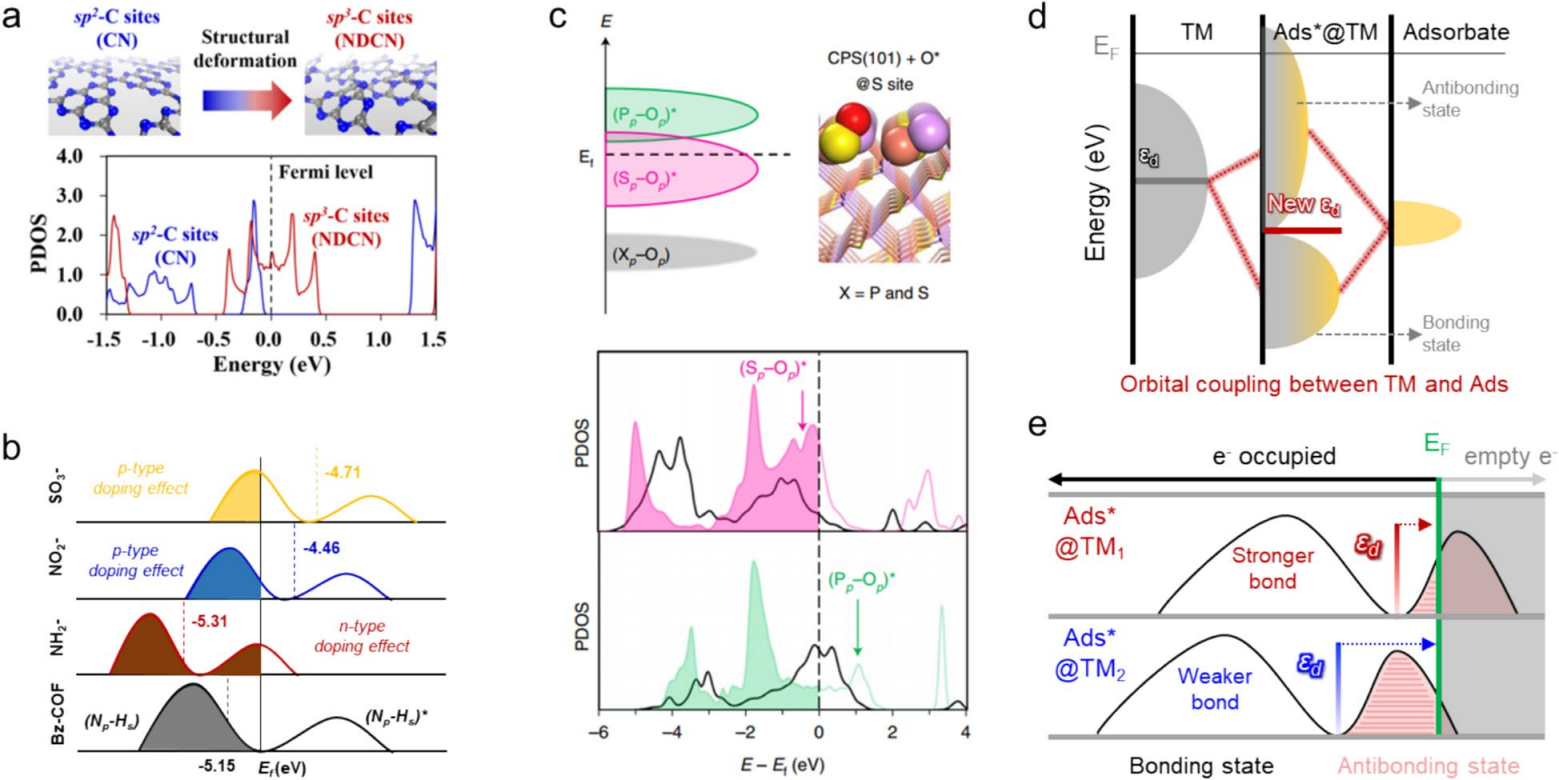
**a** Structural-deformation-induced *sp*^*3*^‑C sites of C_3_N_4-x_ and partial density of states (PDOS) of the *sp*^*2*^‑C and *sp*^*3*^‑C sites in bulk C_3_N_4_ and C_3_N_4-x_, respectively. **b** PDOSs of secondary amines in covalent-organic-frameworks (COF) based on Pt catalysts (Bz-COF, NH_2_-COF, NO_2_-COF, and SO_3_-COF) showing the effects of doping compared to Bz-COF. **c** Schematic showing the antibonding (X_p_–O_p_)* and bonding states (X_p_–O_p_) (where X = S or P) formed between the *p*-orbitals of an O* and the active sites of S or P in copper phosphosulfide (CPS(101)) and the PDOSs of the O*-chemisorbed sites (O*@S and O*@P, in magenta and green) and bare S and P sites (in black) of the CPS(101) surfaces. **d** Schematic showing orbital coupling between an adsorbate and a TM. **e** Schematic comparing the electron-occupied antibonding states above the *d*-band center (*ε*_*d*_) under the Fermi level (E_F_) for TM_1_ (red bar) and TM_2_ (blue bar) in a PDOS, indicative of relative binding strength. (Figure panels are reproduced with permission [90, 108, 109])

In the realm of electronic structures influenced by geometric changes, Wagh et al. provided an insightful analysis into how structural deformations impact the electronic properties and catalytic performance of a N-vacant C_3_N_4_ matrix incorporated with a supramolecular polymer for use in the OER, as shown in Fig. 8a [108]. The study specifically focused on the transition from *sp*^*2*^-hybridized carbon in bulk C_3_N_4_ to *sp*^*3*^-hybridized carbon in N-vacant C_3_N_4-x_. This structural transformation is pivotal because it significantly enhances interactions with key OER intermediates, such as the OOH* radical, which plays a crucial role in overcoming the potential-determining step (PDS) in the OER process. The partial density of states (PDOS) of the *sp*^*3*^-hybridized carbon sites reveals that states more dominantly distributed near the E_F_, which facilitates electron transfer. This proximity to the E_F_ promotes favorable interactions with the OOH* radical, effectively reducing the ${\eta }^{OER}$ required for the OER. These DFT results clearly reveal that modifications in the N-vacant environment directly influence the electronic structure of the material, leading to enhanced overall catalytic efficiency. These findings underscore the usefulness of DOS analyses for connecting electronic structural changes to improvements in catalytic performance, thereby providing critical insight into more-effective electrocatalyst designs and their optimization.

How functional-group modifications impact the electronic structures of organic molecules is a critical aspect of catalysis, particularly for the HER. Park et al. delved into this topic by investigating the HER activities of amine-based covalent organic frameworks (COFs) modified with Pt catalysts, focusing on the influence of various functional groups, such as Bz-, NH_2_-, NO_2_- and SO_3_-, on the COFs. Their approach involved analyzing DOSs associated with the *p*-orbitals of secondary amines, which were identified as H*-binding sites for the HER, as shown in Fig. 8b [109]. The study revealed that the DOS distribution is profoundly affected by the type of functional group attached to the COF. Notably, SO_3_- and NO_2_- were found to induce a *p*-type doping effect that involved shifting the DOS above the E_F_ relative to that of the original Bz-COF group. In contrast, the NH_2_- group exhibited a strong *n*-type doping effect. These changes in electronic structure are attributable to the electron-donating or -withdrawing capabilities of the functional groups, which significantly affect the electronic environment of the COF. Specifically, the electronic configuration resulting from interactions between functional group and COF in NH_2_-COF-modified Pt has filled antibonding orbitals between the nitrogen *p*-orbitals and hydrogen *s*-orbitals. This electronic configuration is particularly favorable for deprotonating H*, thereby facilitating the release of hydrogen and enhancing the overall HER activity. These findings underscore the importance of functional-group selection and modification when tuning the electronic properties of catalysts with the aim of optimizing their performance in specific reactions. Researchers are better able to understand and exploit electronic structural modifications when developing more effective and efficient catalytic systems by deeply analyzing the DOS and its functional-group relationship.

Moreover, as an example of DOSs at different active sites within a system, Shinde et al. explored the OER activity of copper phosphosulfide (CPS(101)) and found enhanced activity at the S site compared to those at the P and Cu sites [90], and identified weak O* binding as the key to lowering the ${\eta }^{OER}$ through FED analysis. Electronic-structural analysis at the O*@S and O*@P sites shows that the antibonding states of (S_*p*_–O_*p*_)^*^ are located near the E_F_, whereas the antibonding states of (P_*p*_–O_*p*_)^*^ are located above the E_F_ (Fig. 8c). Because electrochemical reactions depend on electronic interactions near the E_F_, the presence of (S_*p*_–O_*p*_)^*^ states close to the E_F_ facilitates efficient electron transfer from Cu to S, where partially occupied (S_*p*_–O_*p*_)^*^ states promote the chemisorption of O*, leading to enhanced OER performance. Hence, exploring electronic structures within the catalyst structure using the DOS provides valuable insight into how electronic interactions influence the overall reaction mechanism and catalytic activities.

### *d*-Band theory for adsorbate–catalyst interactions

DOS analyses offer profound insight into how the electronic states near the E_F_ impact the binding strengths of adsorbates on electrocatalysts. Understanding this relationship is crucial as the bond strength between an adsorbate and the catalyst surface is intimately connected to how the antibonding states near the E_F_ are occupied, as described by *d*-band-center theory formulated by Hammer and Nørskov [110]. According to this theory, the *d*-orbitals of the TM and the *s* or *p*-orbitals of the adsorbate couple when an adsorbate binds to the surface of a TM; this interaction is analogous to the formation of chemical bonds described by molecular orbital theory (Fig. 8d). During this bonding process, the *d*-orbitals of the TM spread widely and the position of the *d*-band center (${\varepsilon }_{d})$ shifts due to interactions with the adsorbate. This shift results in partial occupation of the antibonding states below the E_F_ [111]. Since the energy levels of adsorbates in electrocatalytic reactions tend to be consistent across various catalysts; hence, the ε_d_ of a TM in a catalyst becomes essential for understanding the binding affinity of the adsorbate. For instance, an adsorbate interacting with two different TMs binds more strongly to the TM whose ${\varepsilon }_{d}$ is closer to the E_F_ (TM_1_ in Fig. 8e) because antibonding states below the E_F_ are less populated in TM_1_ than in TM_2_ leading to lower electron occupancies in these orbitals, which in turn stabilizes the bond more effectively than in the TM_2_ scenario [112]. By leveraging this theoretical framework, researchers can predict the relative binding affinities of adsorbates on various catalyst surfaces. This capability provides invaluable insight into the catalytic activities of various electrocatalysts, thereby enabling the development and optimization of more effective catalytic systems by strategically modifying their electronic properties in a manner that enhances binding interactions.

**Fig. 9:**
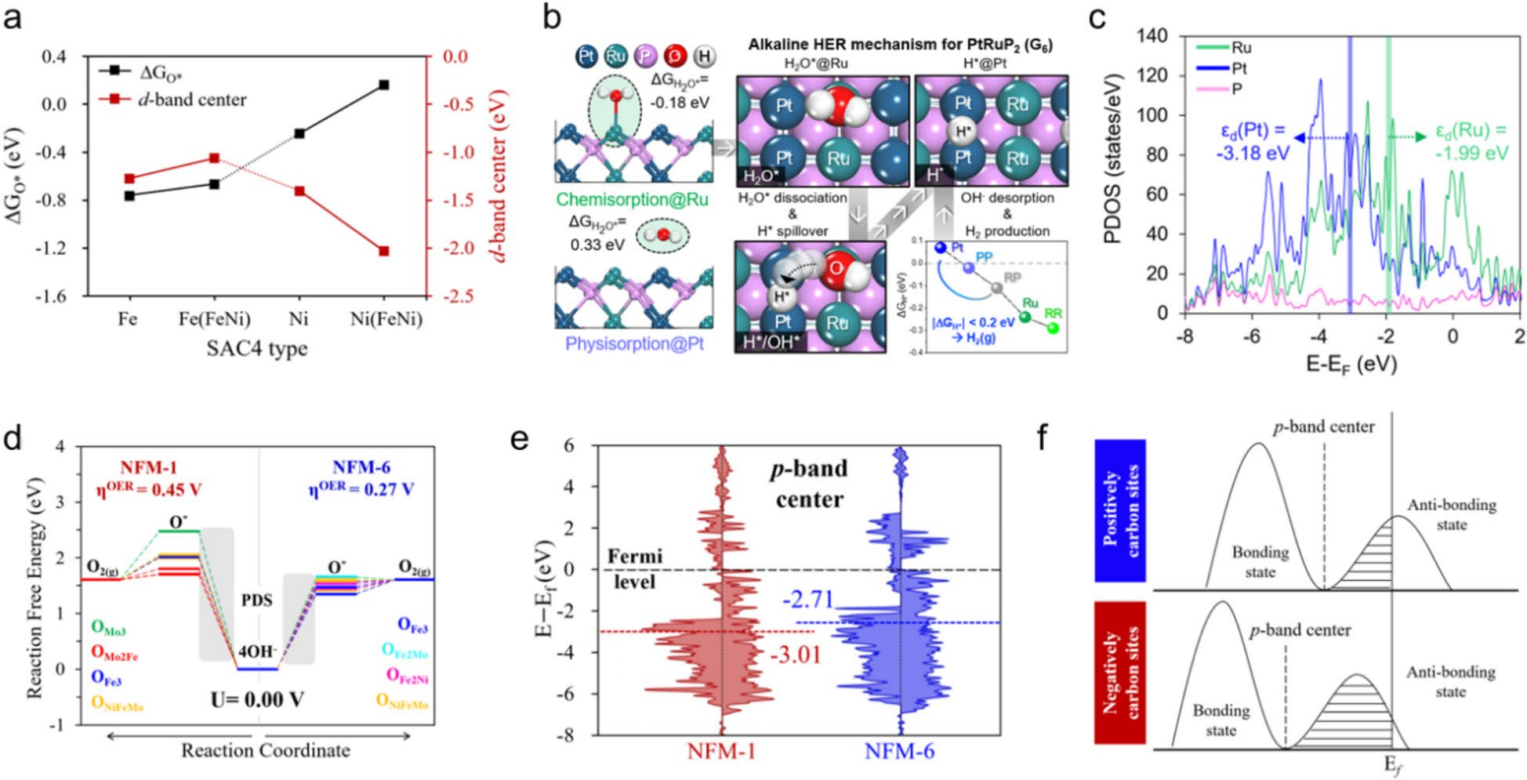
**a** Correlations between *d*-band center (*ε*_*d*_) and O* binding energy ($\Delta {G}_{{O}^{*}}$) for different types of active site on TM@ g-C_3_N_4_ catalysts (TM = Fe, Ni, and Fe/Ni). **b** Schematic showing the alkaline HER mechanism following sequential H_2_O activation/dissociation at Ru-related sites and H_2_ production at Pt-related sites. **c** Partial density of states (PDOSs) of PRP for the ε_d_ values of Ru and Pt active sites. **d** Free energy diagrams (FEDs) for the OER according to the active sites in NFM-1 (Ni_3_Fe_3_Mo_3_(OOH)_9_)) and NFM-6 (Ni_3_Fe_5_Mo_1_(OO)_9_H_7_)) in alkaline media. The shading highlights potential-determining steps (PDSs). **e** PDOSs of the lattice oxygens of NFM-1 and NFM-6 along with their *p*-band centers. **f** Schematics showing the *p*-band center and anti-bonding-state occupations in the partial density of states of positively and negatively curved carbon sites in the schwarzite structure. The dashed lines indicate *p*-band-center values, and E_*f*_ is the Fermi energy level. (Figure panels are reproduced with permission [47, 62, 76, 93])

For example, a theoretically strong correlation exists between the Gibbs free energy for oxygen binding ($\Delta {G}_{{O}^{*}}$) and the *d*-band centers across various SACs on Fe/Ni bimetallic active sites in Fe/Ni@g-C_3_N_4_ in an OER study (Fig. 9a) [76], which revealed that weaker O* binding leads to lower ${\eta }^{OER}$ values, which highlights the significance of the O* binding strength. The authors estimated *d*-band center values for Fe (or Ni)@g-C_3_N_4_ and mixed Fe/Ni@g-C_3_N_4_ SACs to explain the dominance of weaker O* binding at Ni active sites compared to that at Fe sites. The *d*-band center is significantly further shifted from the E_F_ for the Ni active site, which increases anti-bonding-state occupancies and weakens the Ni–O bond when mixed with SACs. In contrast, the *d*-band center of the Fe active sites shifts only slightly closer to the E_F_, resulting in stronger Fe–O bonding. This disparity in *d*-band-center shifts explains the extreme difference in OER activities observed for Ni and Fe active sites, with Ni active sites exhibiting superior OER activities with mixed SACs due to weaker O* binding.

On the other hand, Hong et al. reported exceptional HER performance for the PtRuP_2_ catalyst through superior atomic synergy in the reaction mechanism, in which Ru atoms serve as strong water–adsorption sites and Pt atoms act as hydrogen-adsorption sites with near-optimal binding energies (|$\Delta {G}_{{H}^{*}}$|< 0.2 eV) (Fig. 9b) [47]. In this study, the Ru sites preferentially and strongly binding H_2_O and H*, whereas the Pt sites show relatively weak binding affinities for these intermediates. To understand this difference in binding tendencies, these researchers analyzed the electronic structure of each element on PtRuP_2_ and evaluated the *d*-band centers, which revealed that the *d*-band center of Ru (${\varepsilon }_{d}$(Ru) = –1.99 eV) is closer to the E_F_, indicative of stronger binding, while the *d*-band center of Pt (${\varepsilon }_{d}$(Pt) = –3.18 eV) is further from the E_F_, resulting in a higher antibonding-state electron occupancies and, consequently, lower binding affinities (Fig. 9c). These analyses explain why the Ru and Pt active sites play different roles in promoting the alkaline HER, with Ru facilitating strong water adsorption and Pt enabling optimal hydrogen adsorption. In addition, this valuable descriptor of catalytic activity is not limited to *d*-orbitals, but can also be applied to materials with 2*p*-orbitals, such as oxygen and carbon [113, 114]. Inamdar et al. analyzed the O 2*p*-band center (${\varepsilon }_{p}$) at the active site to understand the difference in OER performance between NFM-1 (Ni_3_Fe_3_Mo_3_(OOH)_9_)) and NFM-6 (Ni_3_Fe_5_Mo_1_(OO)_9_H_7_)) in the Ni_x_Fe_y_Mo_z_ LDH system, which have varied TM compositions and oxidation states [62]. Their analysis revealed that the TMs in NFM-6, particularly at the Fe-rich and Ni-engaged sites, possess higher oxidation states than those in NFM-1, resulting in superior OER performance, as shown in Fig. 9d. The difference in ${\eta }^{OER}$ is ascribable to the varying binding tendencies of O* in the PDS. Accordingly, this group also further analyzed differences in O 2*p*-band centers (Fig. 9e), with the O 2*p*-band center in NFM-6 found to lie higher than that in NFM-1, leading to stronger binding between the active-site oxygens and O* intermediates, which accelerates the OER process and lowers ${\eta }^{OER}$. These findings reveal that the electronic structure of the oxygen in a TM-based oxide material influences ${\eta }^{OER}$ and highlights the importance of the 2*p*-band center as a key descriptor for oxygen-involved reactions.

Furthermore, Seok et al. investigated carbon schwarzite structures characterized by periodic regions of positive and negative curvature (Fig. 9f) [93] and revealed that regions of positive curvature enhance C-2*p*-orbital activation, thereby facilitating more efficient hybridization with H–*s*-orbitals and positively curved regions that exhibit 2*p*-band centers closer to the E_F_. Hence, hydrogen adsorption, with |$\Delta {G}_{{H}^{*}}$| close to 0 eV, is favorable leading to superior HER catalytic performance compared to regions with negative curvature. These findings suggest that electronic structure can be modulated solely by curvature even for a carbon-based material, highlighting the critical role that curvature plays in tuning electronic structures related to catalytic properties. Therefore, applying band-center theory to electrocatalysts greatly enhances our understanding of the complex relationship between adsorption energy and catalytic activity, which provides deeper insight into how to optimize catalyst performance.

## Advanced DFT-simulation concepts

### Influence of adsorbate coverage

**Fig. 10:**
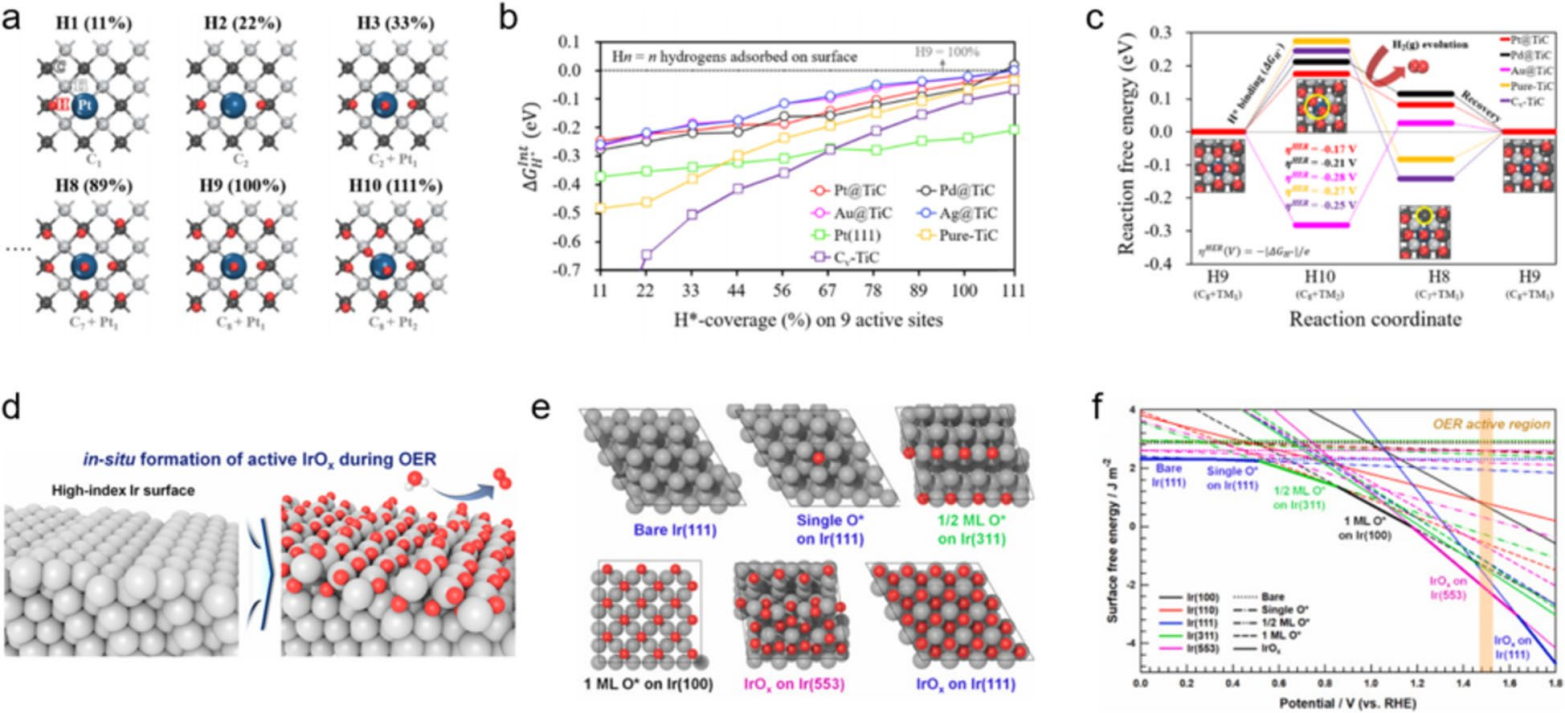
**a** Representative H* configurations of Pt@TiC with increasing H* coverage, referred to as “Hn (C_i_ + Pt_k_),” where i and k represent the number of H* sites on C and Pt, respectively. **b** Calculated integrated H*-adsorption free energies ($\Delta {G}_{{H}^{*}}^{Int}$) for TM@TiC structures as functions of H coverage. **c** FEDs for HERs on H*-passivated TM@TiC surfaces, depicting sequences involving H* binding, H_2_ evolution, and recovery of the H*-passivated surface. **d** Schematic depicting the formation of an active IrO_x_ layer under OER conditions on a high-index Ir surface structure. **e** Top views of the atomic configurations of bare Ir(111), single O* on Ir(111), 1/2 monolayer O* on Ir(311), single monolayer O* on Ir(100), IrO_x_ on Ir(553), and IrO_x_ on Ir(111). **f** Surface free energies as functions of applied potential for Ir(100), Ir(110), Ir(311), and Ir(553) for O*-binding and IrO_x_-formation processes. (Figure panels are reproduced with permission [77, 117])

Adsorbate coverage, particularly H*, O*, and OH*, plays a pivotal role in accurately predicting HER and OER activities under various operational conditions for DFT-simulations involving electrocatalysts [115, 116]. This approach is essential for understanding the interaction dynamics between the adsorbates and the catalyst surface, which directly influences catalytic efficiency. A specific study by Cho et al. investigated the effects of H* passivation in titanium-carbide-based single-atom catalysts (TM@TiC) and explored their HER activities in acidic environments. Comprehensive high-throughput screening identified several stable TM-containing catalysts, including Pt, Pd, Au, and Ag [77]. Their research, which is depicted in Fig. 10a, focused on the preferential binding of H* to carbon sites over TM sites within SACs. This preference led them to investigate the extent of H* coverage and its implications for catalytic activity. Cho et al. quantitatively assessed this by determining the integrated hydrogen binding energy ($\Delta {G}_{{H}^{*}}^{Int}$), which helped them to gauge the thermodynamic feasibility of achieving complete H* coverage on the catalysts. As shown in Fig. 10b, their analysis suggests a H*-passivation layer is potentially formed at full coverage, which is crucial for enhancing HER activity; this passivation layer significantly alters the electronic structure of the TM site, optimizing it as the primary site for hydrogen production. Pt@TiC exhibited the best HER performance among the various TM@TiC catalysts evaluated, as shown in Fig. 10c. This superiority is attributable to the efficient evolution of hydrogen and the dynamic recovery of the H*-passivation layer, which collectively contributes to enhancing catalytic activity. These findings highlight the critical role that adsorbate coverage plays in influencing the electronic properties of catalysts and underscore the importance of detailed theoretical models for predicting and improving the performance of electrocatalysts under practical operating conditions.

Kim et al. explored the role played by surface-O* coverage on Ir nanocrystals (NCs) under acidic conditions in determining OER performance [117]; their study emphasized the significance of concave Ir NCs with high-index surfaces that promote the formation of active IrO_x_ species (O*-covered Ir surface structures) for the OER (Fig. 10d). DFT simulations (Fig. 10e) revealed that O* binding is strongly favored on high-index surfaces ((311) and (553)) compared to low-index surfaces ((100), (110), and (111)), and surface free-energy calculations as functions of applied potential (V_RHE_) identified that the most stable oxidized Ir surfaces occur within a specific potential range, with the fully oxidized Ir(553) surface structure being particularly stable between 1.18 and 1.64 V_RHE_, leading to superior OER activity (Fig. 10f).

### Solvation effects and catalytic performance

**Fig. 11:**
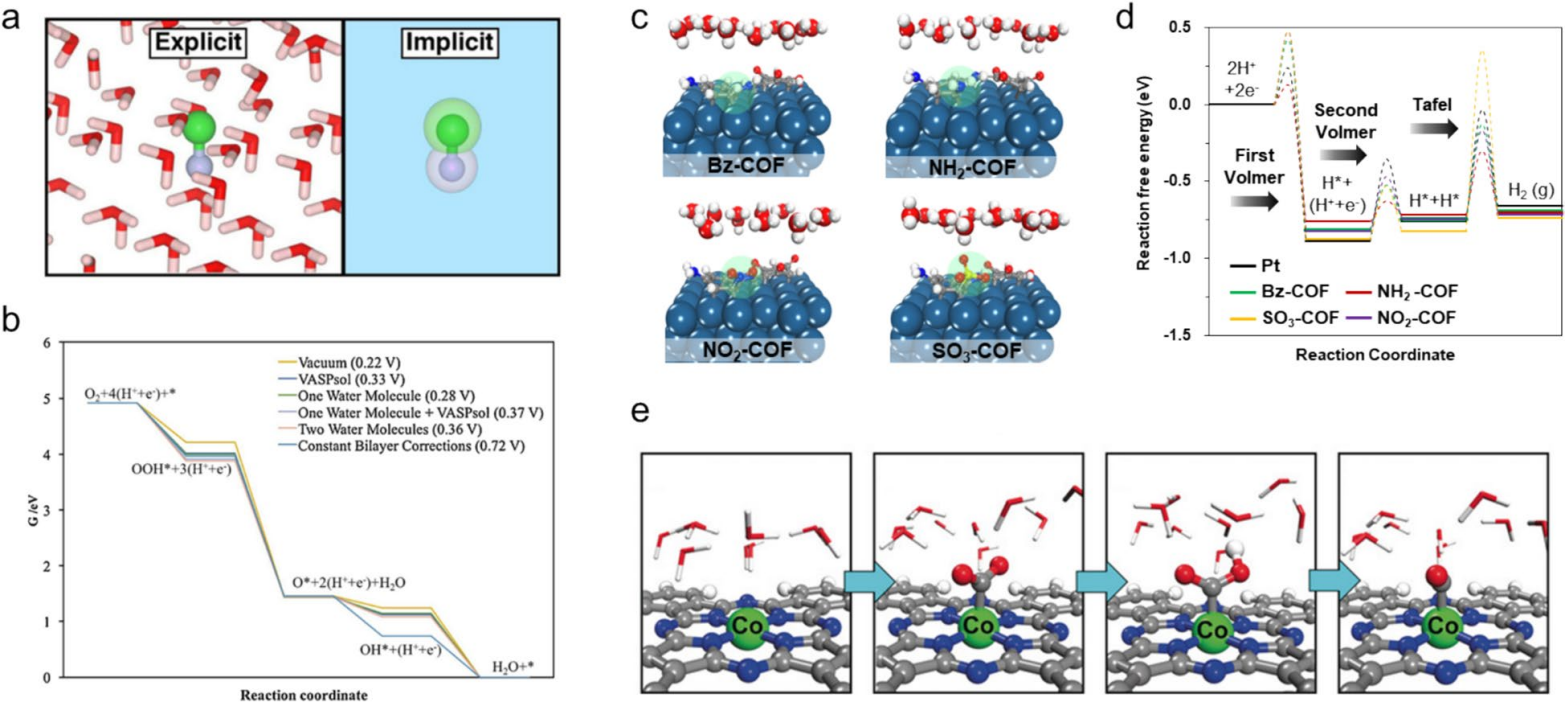
**a** Schematic illustrating explicit and implicit solvation models. **b** FED for the oxygen reduction reaction (ORR) on Pt(111) under different solvation conditions. **c** Illustrating interfacial structures at the Pt(111) surface with a covalent organic framework (COF) and a water layer, using the electrochemical solid–liquid interface (ELSI) model. **d** FED for the HER on COF-modified electrocatalysts, employing the ELSI approach. **e** CO_2_ reduction pathway on CoPc, detailing the adsorption of CO_2_*, formation of COOH*, and adsorption of CO* on the active Co site in the presence of a hydronium ion within the water layer. (Figure panels are reproduced with permission [109, 118, 124, 129])

Using DFT to design advanced HER/OER catalysts requires accurately describing interactions between solvents and catalyst in order to predict catalytic behavior under realistic conditions [118–120]. To that end, two solvation models are generally used: explicit and implicit (Fig. 11a).

(1) The explicit solvation model involves explicitly adding individual solvent molecules to the catalyst model, thereby capturing detailed solute–solvent and solvent–solvent interactions. The approach provides a clear and direct simulation of the role played by the solvent in a catalytic reactions, but is computationally intensive because multiple solvent configurations need to be sampled and large-scale simulations are required. (2) The implicit solvation model treats the solvent as a continuous medium surrounding the solute, which significantly reduces computational demands by averaging the effects of the solvent rather than simulating individual molecules. Although efficient, this approach can overlook specific solute–solvent interactions that are more-effectively captured by explicit models [121–123].

Zhang et al. explored solvation effects in DFT calculations of oxygen reduction reaction (ORR) activity on metal surfaces, with VASPsol used to implicitly model solvation [124]. Although this study focused on the ORR, the overall approach is similar to HER and OER studies and provides valuable insight. While VASPsol was found to significantly improve predicted ORR onset potentials compared to those obtained under vacuum conditions, it tended to underestimate OH* solvation energies on Pt(111) surfaces owing to its inability to effectively capture hydrogen bonding between oxygen species and water (Fig. 11b). This limitation affects the ability to predict catalytic activities across various metal surface structures. While implicit models such as VASPsol are computationally efficient, explicit models are necessary to accurately reproduce solute–solvent interactions.

As an example of the use of an explicit approach, Park et al. investigated the HER activity of Pt catalysts modified with amine-based-COFs bearing various functional groups (Bz-, NH_2_-, NO_2_-, and SO_3_-) with the aim of validating the enhanced HER activity of NH_2_-COF-modified Pt using the electrochemical solid–liquid interface (ESLI) model and a DFT approach. This model calculates the reaction kinetics for proton–electron transfer under acidic conditions by incorporating well-distributed water bilayers (Fig. 11c) [109, 125–128]. The FED calculated using the ESLI approach revealed that NH_2_-COF-modified Pt exhibited the lowest activation barriers in the Volmer and Heyrovsky steps for H_2_ evolution compared with other modified Pt catalysts (Fig. 11d). Considering proton acceptance from the water bilayer, these energy barriers underscore the reliability and accuracy of the results obtained in a solvent-incorporated system.

In addition, Zhang et al. conducted DFT simulations that incorporated explicit solvation effects to investigate the reaction behavior of TM-phthalocyanines (TMPcs, TM = Mn, Fe, Co, Ni, and Cu) in the electrocatalytic CO_2_ reduction (CO_2_RR) reaction [129]. This study included a water monolayer near the TM active sites, which acted as hydronium ions in the solvent (Fig. 11e) providing insight into how different TMs influence the key steps of the CO_2_RR, such as COOH* formation and CO* desorption, while explaining the superior activity of CoPc. These findings emphasize that the introduction of explicit models into electrocatalyst research is crucial because they more-accurately represent solvation effects that are often moderately overlooked.

### Role played by applied potential in a complex catalyst system

The applied potential affects the thermodynamic status of the catalyst in the CHE approach, as observed in the FED. However, this method only adjusts $\Delta G$ after DFT calculations and does not fully capture potential effects at the electrode–electrolyte interface. To better understand catalyst behavior, recent research has focused on potential-dependent models that describe the thermodynamic and kinetic properties of electrocatalysts based on the applied potential.

**Fig. 12:**
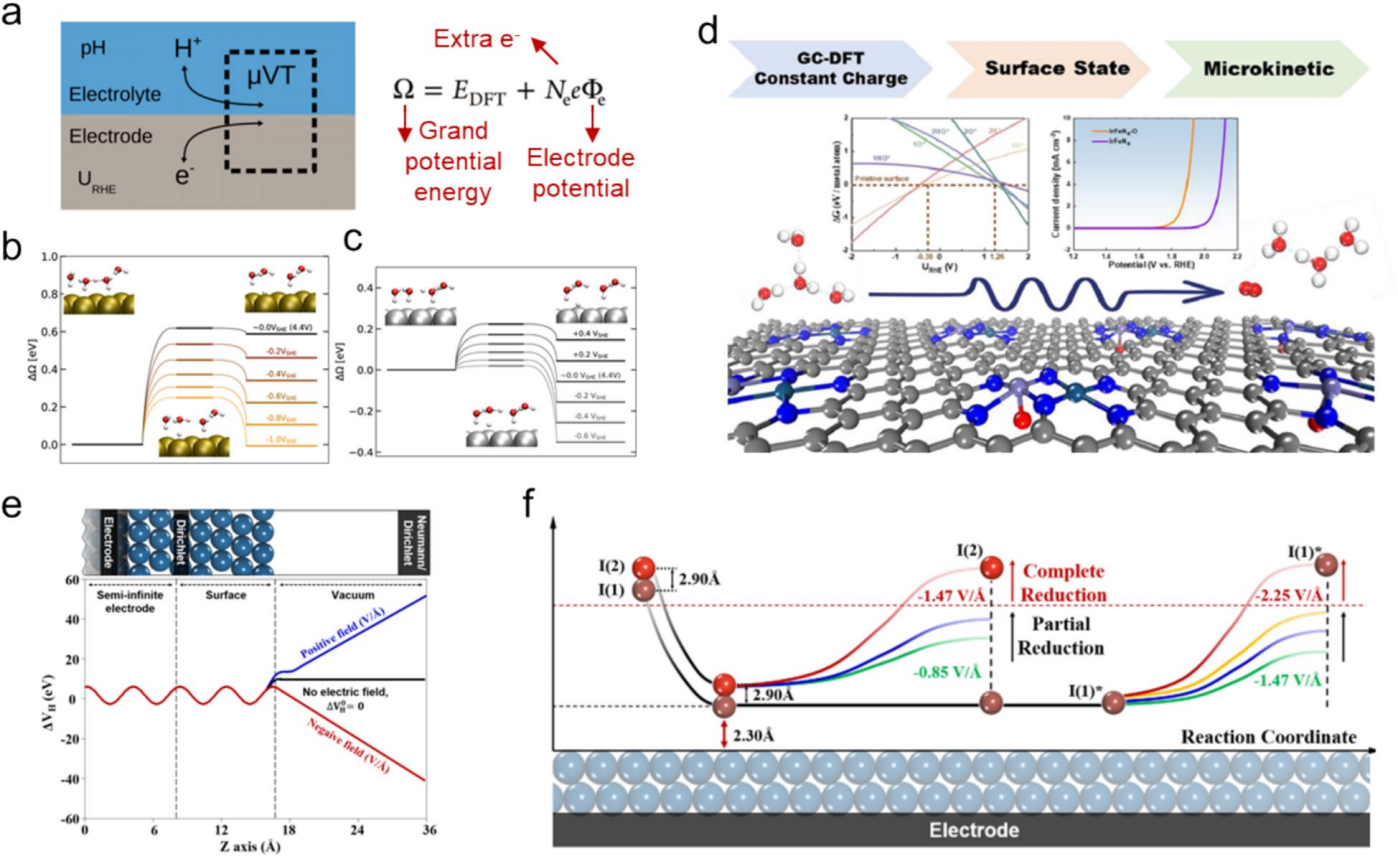
**a** Electrochemical interfacial system within the grand canonical (GC) ensemble, illustrating the use of the grand potential energy for modeling reaction mechanisms at various applied potentials. Grand-potential-energy diagrams relative to the initial state for the Volmer reaction under acidic conditions on **b** Au(111) and **c** Pt(111) surfaces. Voltage values on the right-hand side are shown relative to 4.4 V, which is approximated to be 0 V_SHE_. IS, TS, and FS denote initial, transition, and final states, respectively. **d** Research flowchart depicting how OER performance is evaluated on an O*-preoccupied FeIrN_6_ surface using the GC-DFT approach, which is aimed at determining surface states and exploring catalytic activities. **e** Schematic showing a semi-infinite one-probe system, with average Hartree difference potentials ($\Delta {V}_{H}$) plotted as functions of the z-axis for different external electric fields. A Dirichlet boundary condition is applied at the boundary between the surface and bulk-electrode regions. **f** Schematic depicting the consecutive mechanism associated with iodine reduction, comprising the first reductive dissociation of a vertical I_2_ molecule followed by the reductive desorption of the I(1)* atom in a reductive environment. (Figure panels are reproduced with permission [130, 132, 133, 136])

The grand canonical (GC) approach represents a notable advancement in this area, as illustrated by Hansen et al. (Fig. 12a), offering powerful insight into determining electrochemical behavior under realistic conditions [130, 131]. This approach emphasizes maintaining a constant chemical potential within the model, thereby providing more-precise electrochemical behavior. In this approach, the grand potential energy ($\Omega$) is primarily used as a thermodynamic property, as opposed to the conventional free energy ($\Delta G$), and is derived using the following equation:

32 $$\Omega = E_{DFT} + N_{e} e\Phi_{e}$$

where ${E}_{DFT}$ is the DFT-calculated energy and includes implicit solvation effects, ${N}_{e}$ is the number of extra electrons that follow the reaction pathway, and ${\Phi }_{e}$ is the electrode potential.

Kastlunger et al. used this descriptor to investigate the Volmer step for the HER on Au(111) and Pt(111) surfaces by controlling the applied potential (Fig. 12b, c) [132]. Grand-potential-energy diagrams show how these energies change with potential and reveal that, while the Volmer step is slightly downhill on Pt at 0 V_SHE_, it remains strongly uphill on Au because H* binds more strongly across the Pt potential range. Similarly, Wang et al. employed GC-DFT to study the potential-dependent OER performance of dual Fe-Ir sites on the FeIrN_6_ surface [133]. Their calculations established a Pourbaix diagram at varying potentials that illustrated how the FeIrN_6_ surface states change with potential and pH. They identified a stable O*-preoccupied FeIrN_6_ catalyst under OER conditions and found that the OER is more favorable at the Fe site than at the Ir site, driven by a dual-site O*–O* coupling mechanism for O_2_ evolution. These findings emphasize the importance of potential-dependent effects in DFT calculations for accurately predicting catalytic mechanisms and the surface stabilities of electrocatalysts [134, 135].

Incorporating the effects of electric fields into theoretical models of catalysis is crucial for providing a comprehensive understanding of electron-transfer mechanisms in catalyst systems. Lee et al. introduced a groundbreaking theoretical approach known as the “one-probe and non-equilibrium surface Green’s function (OPNS)” to address the inherent challenges associated with accurately reproducing catalytic reaction mechanisms in electric fields, particularly focusing on the iodine reduction reaction (IRR) on Pt(111) surfaces [136]. Traditional DFT methods often provide a simplified view of surface electronic structures and electron transfer in an electric field induced by applied potentials. However, these simplifications can lead to inaccuracies in predicting real-world catalytic behavior. To overcome these limitations, the OPNS method innovatively couples the surface to an infinite bulk electrode, thereby offering a more-accurate depiction of semi-infinite surfaces in an electric field, as shown in Fig. 12d. Lee et al. provided pivotal insight into the IRR, revealing that it follows a consecutive mechanism driven by the configurational preference of I_2_ molecules in a negative electric field. This finding challenges the traditionally proposed concerted mechanism for the IRR, and highlights the complexities and dynamic nature of catalytic reactions under various electrical conditions (Fig. 12e). The OPNS methodology represents a significant advancement in the simulation and understanding of complex catalytic reactions that are influenced by external electric fields. This approach is expected to play a key role in more accurately predicting and enhancing the performance of catalytic reactions, such as the HER and OER. The OPNS method offers valuable insight that may lead to the development of more effective and efficient catalysts tailored for specific electrochemical applications by more-accurately representing of how electric fields affect catalytic surfaces and electron dynamics.

### Machine learning integrated with DFT in water-splitting research

**Fig. 13:**
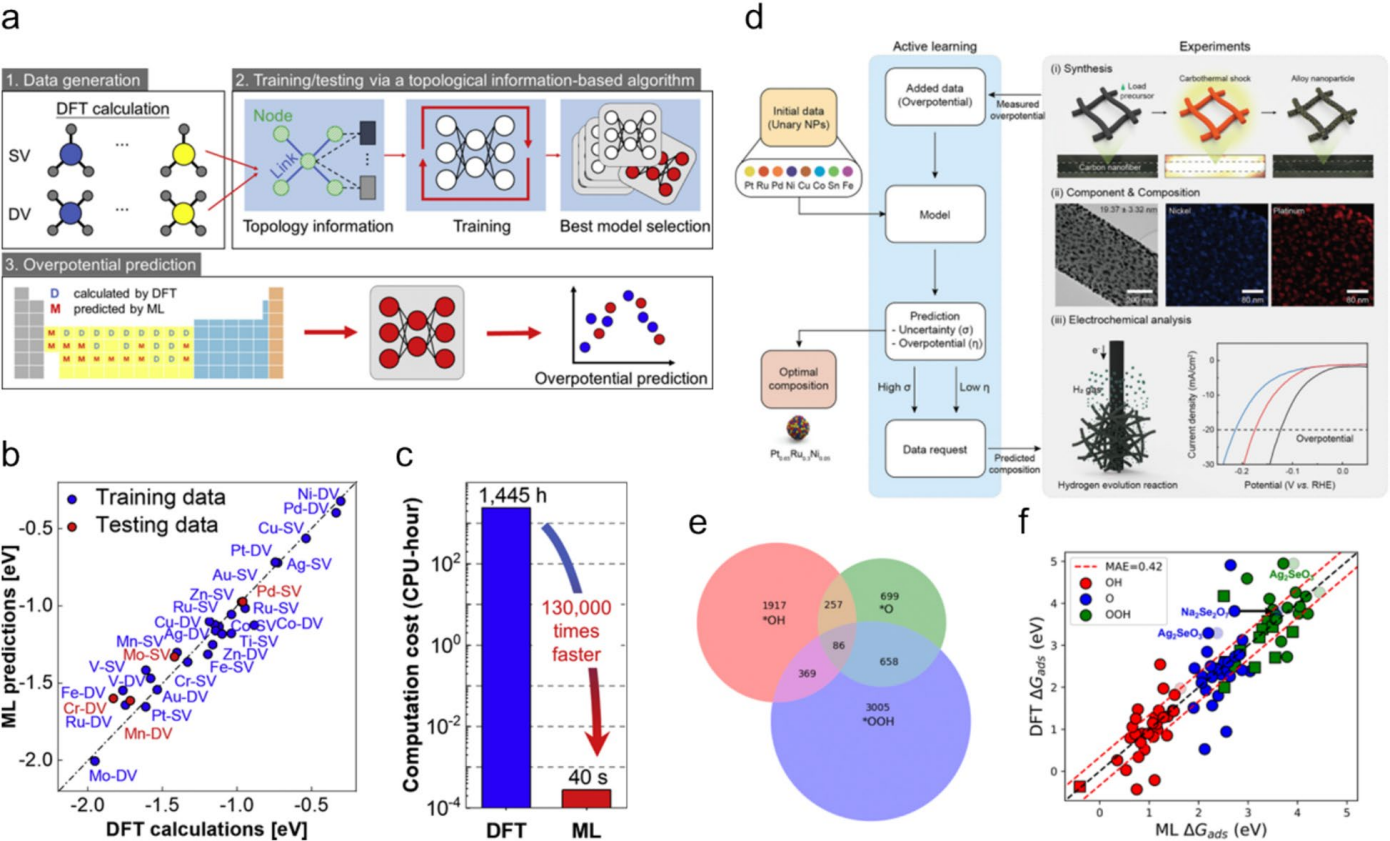
**a** Diagram showing the training strategy used for ML models that involves: (1) data generation, (2) training/testing using a topological-information-based algorithm, and (3) overpotential-prediction workflow. **b** Comparing DFT- and ML-predicted values of *η*^*OER*^. **c** Comparing average computational costs associated with predicting the OER catalytic activity of a transition metal SAC using pure DFT calculations and ML. **d** Overall workflow involved in searching for low-overpotential multi-metallic alloy catalysts: (i) brief schematic depicting multi-metallic alloy-nanoparticle synthesis and corresponding photographic images, (ii) TEM images of well-synthesized alloy nanoparticles, (iii) schematic image and linear sweep voltammetry curves acquired during the electrochemical analysis of fabricated nanoparticles. **e** Surface-intermediate scope for the OER calculated using DFT in the OC22 dataset. Overlaps indicate the numbers of surfaces where different OER intermediates are calculated for the same surface. **f** DFT-calculated reaction energies plotted against the corresponding ML predicted quantities of 33 stable compounds from the OC 22 dataset; square data points indicate desorption or dissociation of the intermediate. (Figure panels are reproduced with permission [139, 144, 145])

Recent breakthroughs in machine learning (ML) have significantly advanced its use in HER/OER catalyst-design applications. Integrating ML with DFT has led to notable success, particularly for the high-throughput screening of efficient catalyst designs, which has traditionally been challenging owing to the high computational costs associated with DFT calculations. However, ML has accelerated this process, enabling a broad sampling space that includes various catalyst types, such as 2D and 3D materials, to be explored. For example, Xiaoxu et al. applied an AdaBoost ensemble learning model trained using five selected descriptors to identify 188 stable HER catalysts from 2520 2D MXene candidates, with 110 MXene 2D alloys found to exhibit superior activity to Pt [137]. Sun et al. used a support vector regression (SVR) model to predict the H* adsorption energy of 271 MBene and MXene systems [138], which was validated with 25 additional MBene alloys. A subsequent round of DFT screening revealed that Co_2_B_2_ and Mn/Co_2_B_2_ are ideal HER catalysts. Actively researched materials, such as SACs, were also screened using topological-information-based algorithms combined with neural networks (Fig. 13a), which revealed a strong correlation with OER catalytic activity across all transition-metal SACs (Fig. 13b), while accelerating computation speeds by up to 130,000-fold compared to those of traditional DFT calculations (Fig. 13c) [139]. The accuracy of these neural network predictions was validated through benchmarking against experimental results, highlighting their reliability and strong agreement with measured OER activities.

More recently, Fang et al. demonstrated the effectiveness of gradient boosting regression combined with DFT to evaluate the OER performance of 729 DACs, which identified 10 materials with activities superior to that of RuO_2_ [140]. Additionally, metal oxides, particularly perovskite oxides, have garnered significant attention and have been explored using per-site graph-neural networks (GNNs). In this study, the GNN autonomously learned site-dependent descriptors from structures to accurately predict the binding energies of OER intermediates without requiring handcrafted features [141]. A variety of promising perovskite oxide materials for the OER were identified from a pool of over 10,000 structures using this method. ML has been used in high-throughput screening to construct Pourbaix diagrams of Pt NPs with diameters of up to 4.8 nm [142]. In this work, the authors developed a bond-type embedded crystal graph convolutional neural network (CGCNN) model to predict the stabilities of differently sized Pt NPs in electrochemical environments with varying O* and OH* coverages. This approach effectively bridges the gap between traditional theoretical calculations and experimental observations.

Although ML has demonstrated significant potential for advancing water-splitting catalyst research, it still faces notable limitations, with data availability being the most prominent issue. Well-constructed and diverse training datasets are crucial for enhancing the predictive capabilities and generalizabilities of ML models. Training datasets typically originate from three main sources: DFT calculations, existing literature (including experimental data), and open databases. However, data from high-throughput DFT calculations are expensive, and data from the literature or large-scale experiments are either rare or inconsistent. A promising solution has emerged in recent years to address these challenges, which involves incorporating active learning into the training process to augment the data. Tran et al. developed a flexible framework integrated with active learning to automate selecting and executing DFT calculations and successfully identified 14 promising HER catalyst candidates from 1499 intermetallic materials [143]. This ML framework was conducted through iterative training cycles, where predicted results were cross-referenced with DFT-computed outcomes to refine the model's accuracy and reliability, demonstrating its capability to effectively identify high-performing catalysts within a vast compositional space. The top candidates undergo additional validation through experimentation. Similarly, Kim et al. combined active learning with experimental methods to optimize the compositions of high entropy alloys (HEAs) for the HER [144]. The mole fractions of the metal precursors required to synthesize HEA nanoparticles was proposed during each iteration of the active learning loop, which ultimately identified Pt_0.65_Ru_0.30_Ni_0.05_ as the optimal catalyst for the HER (Fig. 13d). Data from open databases also serve as a critical resource because they quickly provide multiple precomputed properties of various materials, which was leveraged in a recent study that used a GNN to screen OER catalysts from the Open Catalyst 2022 (OC22) database [145]. Using valuable DFT-calculated OC22 data (Fig. 13e), researchers used the GemNet-OC model to interpolate key data points and determine the OER overpotential. Twenty-two promising catalyst candidates for the OER in bulk systems and 68 candidates in nanoscale regimes were identified after validating the accuracy of the interpolated database (Fig. 13f).

In addition to constructing data for the training process, the applicability of ML to water-splitting research is limited by the challenge of extracting physical insight from ML models. To date, the most successful ML applications in catalysis have relied on black-box models that operate in large feature spaces, making it difficult to interpret the results into meaningful mathematical formulas for the training dataset [146]. To address this issue, recent studies have explored the use of symbolic regression methods that use mathematical operators to combine input features with the aim of discovering simple mathematical expressions that predict target properties based on these features. For example, μ/t is an activity descriptor for the OER on perovskite oxides, where μ and t represent the octahedral and tolerance factors respectively, constructed using symbolic regression methods. This descriptor enabled accelerated searching for new perovskite catalysts that led to four new perovskites with excellent experimental activities being proposed [147]. Integrating ML techniques into HER/OER research has significantly advanced the discovery and optimization of efficient catalyst materials. This integration has enabled the identification of novel candidates with superior performance characteristics by ensuring that ML predictions are rigorously validated through robust training methodologies and independent dataset evaluations, demonstrating their reliability and applicability for actual catalyst design.

## Conclusion

In this review, we discussed recent advances in electrocatalyst research, focusing on the applications of DFT to hydrogen evolution reaction (HER) and oxygen evolution reaction (OER) water-splitting catalysts. We highlighted the essential role played by DFT in identifying promising catalysts and elucidating their atomic-level mechanisms, particularly in nanostructured systems. Importantly, well-established CHE-FED methods provided valuable insight into catalytic activities in HER and OER models, facilitating the development of catalysts with lower precious-metal contents. Furthermore, we emphasized the importance of understanding electronic structures, including density of states (DOS) and *d*-band theory, as fundamental for enhancing catalytic performance. DFT studies have shown that electronic structures are sensitive to geometric modifications, such as atomic doping and heterostructure formation, which significantly affect the overall catalytic activity. Moreover, advanced DFT approaches that include adsorbate coverage, solvation models, and electric-field effects, have improved simulation accuracies under more realistic conditions. Finally, integrating machine learning with DFT has accelerated catalyst discovery through the high-throughput screening of potential candidates. As computational techniques continue to evolve, DFT remains an indispensable tool for driving breakthroughs in sustainable hydrogen production using water-splitting technologies.

## Data Availability

The review is based on the published data and data sources upon which conclusions can be found in the reference list.
